# New insights into the heterogeneity of Th17 subsets contributing to HIV-1 persistence during antiretroviral therapy

**DOI:** 10.1186/s12977-016-0293-6

**Published:** 2016-08-24

**Authors:** Vanessa Sue Wacleche, Jean-Philippe Goulet, Annie Gosselin, Patricia Monteiro, Hugo Soudeyns, Rémi Fromentin, Mohammad-Ali Jenabian, Shant Vartanian, Steven G. Deeks, Nicolas Chomont, Jean-Pierre Routy, Petronela Ancuta

**Affiliations:** 1 Département of Microbiologie, Infectiologie Et immunologie, Faculté de Médecine, Université de Montréal, Montreal, QC Canada; 2 Centre de recherche du CHUM, 900 rue Saint-Denis, Tour Viger, R09.416, Montreal, QC H2X 0A9 Canada; 3 Caprion, Montreal, QC Canada; 4 Unité d’immunopathologie virale, Centre de recherche du CHU Sainte-Justine, Montreal, QC Canada; 5 Département des Sciences Biologiques, Université du Québec à Montréal, Montreal, QC Canada; 6 Department of Medicine, University of California San Francisco, San Francisco, CA USA; 7 Chronic Viral Illness Service and Research Institute, McGill University Health Centre, Montreal, QC Canada; 8 Division of Hematology, McGill University Health Centre, Montreal, QC Canada

**Keywords:** Human, Th17, CCR6, CCR4, CXCR3, HIV reservoirs, ART

## Abstract

**Background:**

Th17 cells are permissive to HIV-1 infection and their depletion from the gut of infected individuals leads to microbial translocation, a major cause for non-AIDS co-morbidities. Most recent evidence supports the contribution of long-lived Th17 cells to HIV persistence during antiretroviral therapy (ART). However, the identity of long-lived Th17 cells remains unknown.

**Results:**

Here, we performed an in-depth transcriptional and functional characterization of four distinct Th17 subsets and investigated their contribution to HIV reservoir persistence during ART. In addition to the previously characterized CCR6^+^CCR4^+^ (Th17) and CCR6^+^CXCR3^+^ (Th1Th17) subsets, we reveal the existence of two novel CCR6^+^ subsets, lacking (double negative, CCR6^+^DN) or co-expressing CXCR3 and CCR4 (double positive, CCR6^+^DP). The four subsets shared multiple Th17-polarization markers, a fraction of cells proliferated in response to *C. albicans,* and exhibited lineage commitment and plasticity when cultured under Th17 and Th1 conditions, respectively. Of note, fractions of CCR6^+^DN and Th17 demonstrated stable Th17-lineage commitment under Th1-polarization conditions. Among the four subsets, CCR6^+^DN expressed a unique transcriptional signature indicative of early Th17 development (IL-17F, STAT3), lymph-node homing (CCR7, CD62L), follicular help (CXCR5, BCL6, ASCL2), and self-renewal (LEFI, MYC, TERC). Cross sectional and longitudinal studies demonstrated that CCR6^+^DN cells were the most predominant CCR6^+^ subset in the blood before and after ART initiation; high frequencies of these cells were similarly observed in inguinal lymph nodes of individuals receiving long-term ART. Importantly, replication competent HIV was isolated from CCR6^+^DN of ART-treated individuals.

**Conclusions:**

Together, these results provide new insights into the functional heterogeneity of Th17-polarized CCR6^+^CD4^+^ T-cells and support the major contribution of CCR6^+^DN cells to HIV persistence during ART.

**Electronic supplementary material:**

The online version of this article (doi:10.1186/s12977-016-0293-6) contains supplementary material, which is available to authorized users.

## Background

The Th17 cells represent a subset of CD4^+^ T-cells that act as the first line of defense against pathogens at barrier surfaces [1]. Th17 cells are defined by the production of unique effector cytokines (IL-17A, IL-17F, IL-21, IL-22, IL-26, CCL20) under the transcriptional regulation of multiple lineage-specific transcription factors (IRF4, BATF, STAT3, RORγt) [2, 3]. Their anatomic localization and effector functions, together with their unique developmental plasticity, position Th17 cells at the very core of the immune system [2, 4]. CCR6 is a well-established marker for Th17 cells [5, 6]. CCR6 mediates recruitment into specific tissues including Peyer’s patches (CCR6 [7]), skin [8] and brain [9]. Although not all CCR6^+^ T-cells produce IL-17A upon stimulation ex vivo, the majority of these cells are prone to acquire Th17 effector functions [10]. The co-expression of CCR6 and CCR4 identifies cells producing IL-17A (Th17 profile) specific to *Candida albicans* and *Staphylococcus aureus* [5, 11], while CCR6^+^CXCR3^+^ cells produce both IL-17A and IFN-γ (Th1Th17 profile) in response to *Mycobacterium tuberculosis* or upon polyclonal stimulation [ [5], [12] ]. These advances in the identification of surface markers for functionally distinct CD4^+^ T cell subsets proved to be instrumental for understanding the contribution of Th17 cells to human pathologies including rheumatoid arthritis [13], multiple sclerosis [9], cancer [14], and HIV-infection [15, 16].

The existence of functionally distinct IL-17A-producing CD4^+^ T-cells cells was originally reported in the context of autoimmunity, with CCR6^+^CXCR3^+^Th1Th17 and CCR6^+^CCR4^+^Th17 cells being considered pathogenic and non-pathogenic, respectively [3, 17]. This discovery led to the identification of molecular signatures associated with Th17 pathogenicity in mice [18–20] and most recently in humans [21]. In contrast, during HIV-1 infection, we previously demonstrated that both CCR6^+^CCR4^+^Th17 and CCR6^+^CXCR3^+^Th1Th17 cells are pathogenic since they are permissive to viral infection in vitro, carry integrated HIV-DNA in vivo, and their frequency is significantly reduced in HIV-infected individuals, including those with undetectable plasma viral load under antiretroviral therapy (ART) [15]. Considering the fact that IL-17A plays a critical role in maintaining epithelial barrier integrity at intestinal level [22, 23], the depletion of Th17 and Th1Th17 cells from gut-associated lymphoid tissues (GALT) is considered as a major cause for microbial translocation, chronic immune activation and occurrence of non-AIDS co-morbidities in HIV-infected individuals [24]. Thus, features of Th17 pathogenicity are unique in the context of HIV infection. In addition, long-lived Th17 cells exist and were reported to promote cancer progression [25]. The possibility that long-lived Th17 cells contribute to HIV reservoir persistence under ART, as supported by recent findings by our group (Gosselin et al, unpublished observations) and others [26], adds to the complexity of Th17 pathogenicity concept and position these cells as a major barrier for HIV eradication.

In this study, we used a systems biology approach an revealed phenotypic, functional and transcriptional features of two previously uncharacterized human CD4^+^ T-cell subsets expressing the Th17 marker CCR6 and lacking or co-expressing the homing receptors CCR4 and CXCR3: CCR4^−^CXCR3^−^ (double negative; CCR6^+^DN) and CCR4^+^CXCR3^+^ (double positive; CCR6^+^DP). Our results provide new insights into the diversity of Th17 subsets during homeostasis and HIV-1 infection, thus adding a novel piece of complexity to the recent understanding of Th17 functional heterogeneity and clonotype sharing in humans [27]. We reveal that CCR6^+^DN are distinguished from Th17, Th1Th17 and CCR6^+^DP by their expression of markers of early Th17 development, lymph node trafficking, follicular help and self-renewal. We also demonstrate that CCR6^+^DN represent the most predominant Th17 subset in the blood and lymph nodes of HIV-infected ART-treated individuals and carry replication-competent integrated HIV-DNA. These findings support the newly emerged concept that HIV takes advantage of the long-lived properties of specific Th17 subsets [25, 28, 29] to ensure its persistence during ART. Thus, permissiveness to HIV-DNA integration compatible with survival represents a new previously unrecognized feature of pathogenic Th17 cells during HIV infection

## Results

### Two novel subsets of memory CCR6^+^ T-cells exhibit Th17-features

**Fig. 1:**
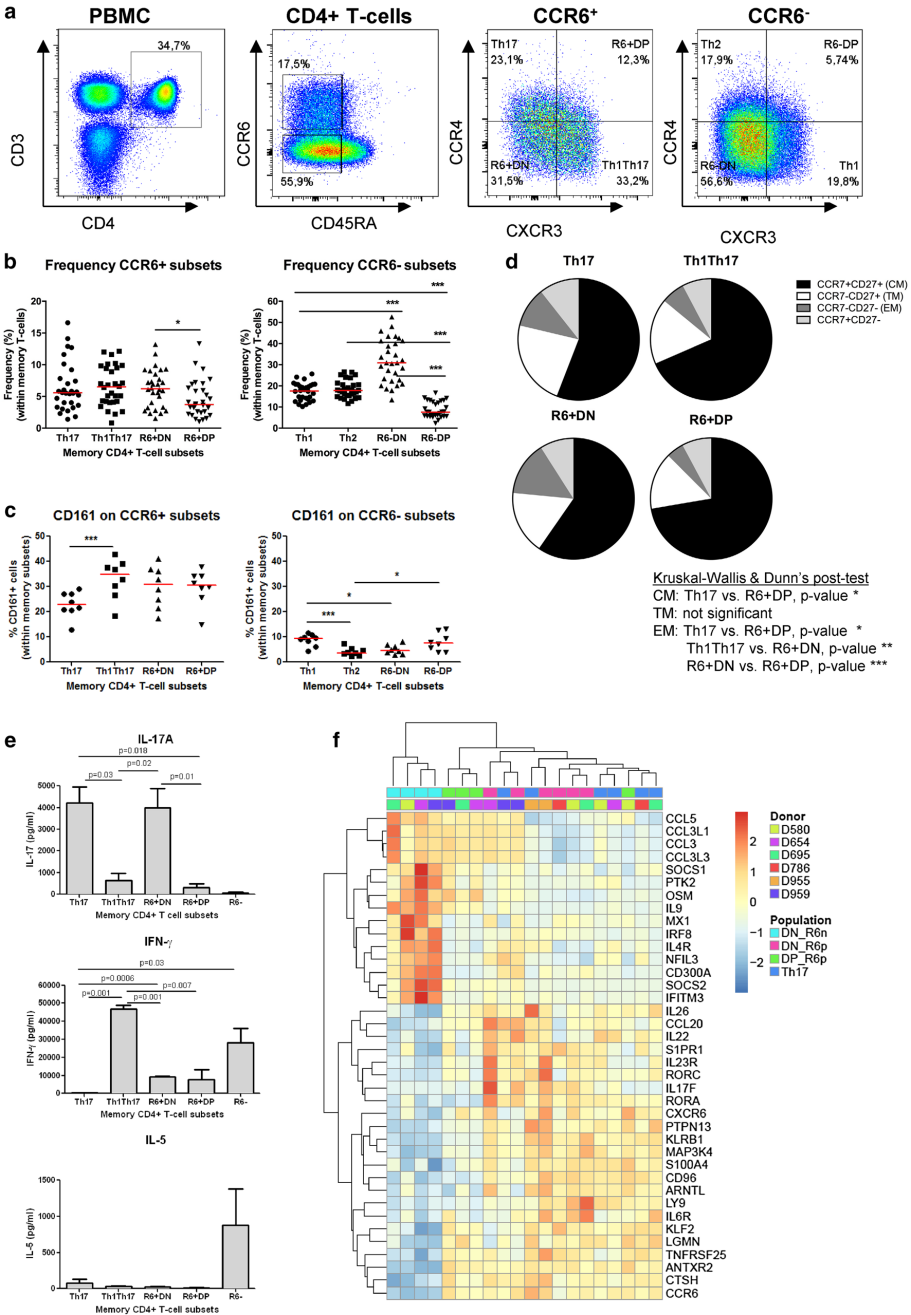
Two new subsets of memory CD4^+^ T-cells express Th17 lineage markers. **a** Memory CD4^+^ T-cells (CD3^+^CD4^+^CD45RA^−^) isolated from the peripheral blood of HIV-uninfected individuals were analyzed for their differential expression of CCR6, CCR4, and CXCR3. CCR6^+^ subsets included: CCR4^+^CXCR3^−^ (Th17), CCR4^−^CXCR3^−^ (double positive, CCR6^+^DP), CCR4^+^CXCR3^+^ (double negative, CCR6^+^DN), and CCR4^−^CXCR3^+^ (Th1Th17). CCR6^−^ subsets included: CCR4^+^CXCR3^−^ (Th2), CCR4^+^CXCR3^+^ (CCR6^−^DP), CCR4^−^CXCR3^−^ (CCR6^−^DN) and CCR4^−^CXCR3^+^ (Th1). Shown is the frequency of CCR6^+^ and CCR6^−^ subsets (**b**; n = 30) and their expression of CD161 (**c**; n = 8). Each symbol represents a distinct subject. Paired t-Test *p*-values are indicated on the figures. *Horizontal bars* indicate median values. **d** Shown are median frequencies of central (CM, CCR7^+^CD27^+^), transitional (TM, CCR7^−^CD27^+^) and effector (EM, CCR7^−^CD27^−^) memory cells per CCR6^+^ subset (n = 10). **e**–**g** FACS-sorted memory subsets (S1 Figure) were stimulated via CD3/CD28 for 4 days. **e** The production of the lineage-specific cytokines IL-17A, IFN-γ, and IL-5 was quantified by ELISA. Shown are results (mean ± SEM) on matched Th17, Th1Th17, CCR6^+^DN, CCR6^+^DP, and CCR6- (n = 3–7). Paired *t* Test *p*-values are indicated on the *figures*. **f** Transcriptional profiling were generated using the HumanHT-12 v4 Expression BeadChip; (Illumina). The heat map depicts differential expression of well-established Th17 and Th1 transcripts (identified as being up/down regulated in Th17 *versus* CCR6^−^DN, *p* value <0.05, fold change cut-off 1.3) in matched CCR6^−^DN versusCCR6^+^DN, CCR6^+^DP and Th17 (n = 4–6; up-regulated genes in red; down-regulated genes in *blue*)

Differential expression of CCR4 and CXCR3 identifies four memory (CD45RA^−^) CCR6^+^ and four CCR6^−^ subsets. The following four subsets were previously demonstrated to be enriched in cells with specific polarization features: CCR6^+^CCR4^+^ (Th17) and CCR6^+^CXCR3^+^ (Th1Th17), CCR6^−^CXCR3^+^ (Th1), and CCR6^−^CCR4^+^ (Th2) [5, 15, 27]. While acknowledging the fact that these subsets are heterogeneous, for simplicity, these subsets are typically identified as Th17, Th1Th17 (or Th1*), Th1, or Th2 cells [5, 12, 21, 27]. We previously demonstrated that Th17 and Th1Th17 subsets are highly permissive to R5 and X4 HIV-1 infection in vitro [15]. Among CCR6^+^ T-cells, two subsets remain uncharacterized in terms of polarization profile and contribution to HIV-1 pathogenesis: CCR4^−^CXCR3^−^ (double negative; CCR6^+^DN) and CCR4^+^CXCR3^+^ (double positive; CCR6^+^DP) (Fig. 1a). In HIV-uninfected individuals, the frequency of blood CCR6^+^DN was similar to that of Th17 and Th1Th17, while the frequency of CCR6^+^DP was slightly lower (Fig. 1b). The CCR6^−^ population also included two previously uncharacterized subsets, CCR6^−^DN and CCR6^−^DP, with CCR6^−^DN being the most predominant (Fig. 1a, b). CCR6^+^DN and CCR6^+^DP expressed the Th17 marker CD161 [30] at levels higher and similar compared to Th17 and Th1Th17, respectively (Fig. 1c). The frequency of cells expressing CD161 was relatively low on CCR6^−^ subsets (Fig. 1c). CCR7 and CD27 identify distinct memory subsets [31, 32]: CCR7^+^CD27^+^ (CM, central memory), CCR7^−^CD27^−^ (EM, effector memory), CCR7^−^CD27^+^ (TM, transitional memory) and CCR7^+^CD27^−^ subset. CM predominated over EM and TM in all CCR6^+^ subsets, with the highest % of CM in Th1Th17 and CCR6^+^DP (Fig. 1d).

For subsequent functional studies, sufficient numbers of highly pure CCR6^+^ and CCR6^−^ cells with differential CCR4 and CXCR3 expression were sorted by flow cytometry from large numbers of PBMCs (10^9^) obtained by leukapheresis (Additional file 1: S1 Figure) from HIV-uninfected individuals. To determine the polarization profile of CCR6^+^DN and CCR6^+^DP, the expression of lineage-specific cytokines was measured in FACS-sorted cells exposed to CD3/CD28 triggering. Compared to Th17 and Th1Th17, CCR6^+^DN and CCR6^+^DP produced high and low levels of IL-17A, respectively. In contrast to Th17, CCR6^+^DN and CCR6^+^DP produced IFN-γ but at significantly lower levels compared to Th1Th17; as expected, the Th2 cytokine IL-5 was produced by CCR6^−^ but not CCR6^+^ cells (Fig. 1e).

To further investigate the Th17 polarization profile of CCR6^+^DN and CCR6^+^DP, their transcriptome was compared to that of Th17 and CCR6^−^DN using the Illumina technology. The list of 1020 transcripts differentially expressed in CCR6^+^DN versus CCR6^−^DN (adjusted *p* value >0.05; FC cut-off 1.3), included well-established markers for Th17 (IL-17F, KLRB1/CD161, CCL20, KLF2, CTSH, IL-22, CCR6, RORA, IL-23R, RORC, PTPN13, IL-26, S100A4, ARNTL, IL-6R) and Th1 lineages (CCL3L3, CCL3, CCL3L1, CCL5, CD300a), potentially new Th17 markers (CD96, LGMN, ANTXR2, CXCR6, MAP3K4, TNFSF25, LY9, S1PR1), together with negative regulators of Th17 polarization (IL-9, IFITM3, OSM, IRF8, MX1, NFIL3, PTK2, IL4R, SOCS1) (Fig. 1f). Clustering analysis of these transcripts grouped all CCR6^+^ subsets together, indicative of their common Th17 polarization. These results identify CCR6^+^DN and CCR6^+^DP as two novel Th17 subsets, with CCR6^+^DN producing high levels of IL-17A and being as preponderant as the previously described Th17 and Th1Th17 in the peripheral blood of healthy humans [5].

### Th17-polarized CCR6^+^DN and CCR6^+^DP subsets exhibit unique transcriptional profiles

**Fig. 2:**
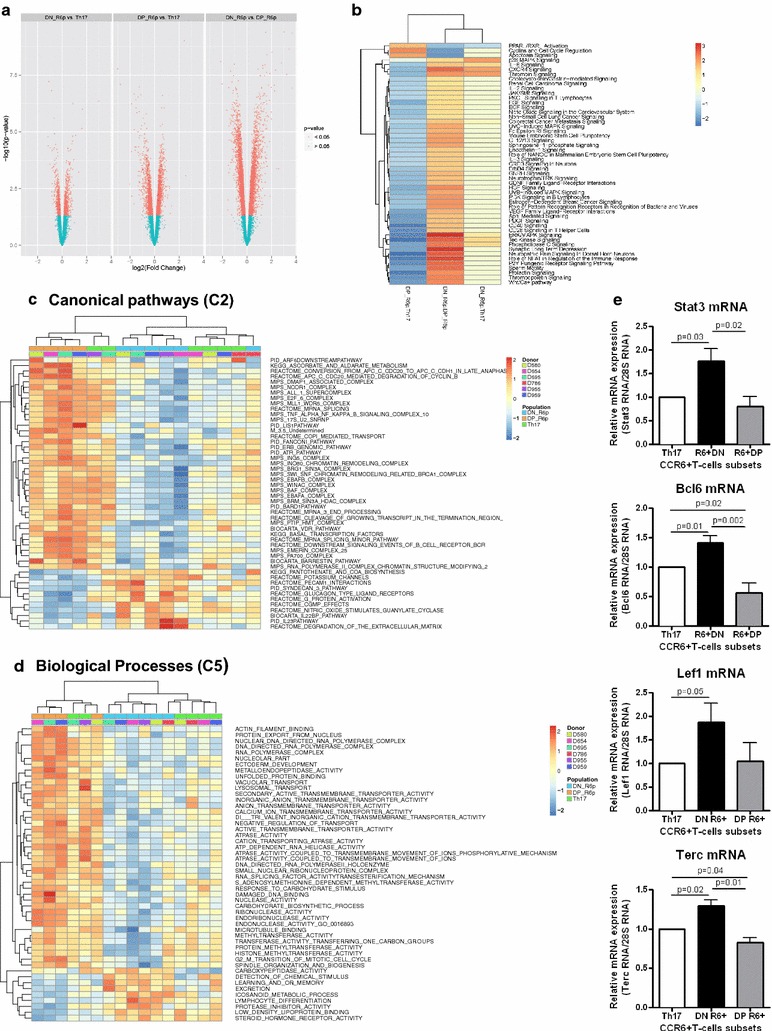
CCR6^+^DN and CCR6^+^DP cells express unique transcriptional signatures. Genome-wide transcriptional profiling was performed on sorted matched Th17, CCR6^+^DN, and CCR6^+^DP (n = 4–6) isolated from the peripheral blood of HIV-uninfected individuals, as in Fig. 1f. Shown are (**a**) volcano representation of differentially expressed probe sets in CCR6^+^DN versus Th17, CCR6^+^DP versus Th17, and CCR6^+^DN versus CCR6^+^DP (depicted in *red*: *p* values <0.05 and fold change cut-off 1.3). **b**, **d** For the same contrasts, shown are heat maps depicting top modulated pathways identified using *Ingenuity Pathway Analysis* (IPA) (**b**) and *Gene Set Variation Analysis* (GSVA) canonical pathways (**c**) and biological functions (**d**). Genes up and down regulated in different subsets are represented in red and blue, respectively. **e** Expression of STAT3, BCL6, LEF1, and TERC mRNA was quantified by real-time RT-PCR (mean ± SEM; n = 3–4). Paired t-Test *p*-values are indicated on the *figures*

Further analysis of transcriptional profiles identified 344, 1407 and 3148 probe-sets differentially expressed by CCR6^+^DN versus Th17, CCR6^+^DP versus Th17, and CCR6^+^DN versus CCR6^+^DP (*p*-value <0.05; FC cut-off 1.3), respectively (Fig. 2a). Differences in gene expression in CCR6^+^DN versus Th17 (13 up; 13 down) were also found inferior to differences in CCR6^+^DP versus Th17 (368 up; 357 down) and CCR6^+^DN versus CCR6^+^DP (1537 up; 1417 down), when transcripts with adjusted p-values <0.05 were considered (Additional file 2: Table S1, data not shown). These results reveal the transcriptome of CCR6^+^DN is similar to that of Th17, but highly distinct from that of CCR6^+^DP. Of note, IL-17F, a cytokine associated with early Th17 development [33], was included among the top up-regulated transcripts in CCR6^+^DN versus Th17 (FC: 7.8) and CCR6^+^DN versus CCR6^+^DP (FC: 12.8) (Additional file 2: Table S1). In contrast, LMNA, a senescence marker [34], was included among the top down-regulated genes in CCR6^+^DN versus Th17 (FC: −2.2) and CCR6^+^DN versus CCR6^+^DP (FC: −4).

To extract functional meaning from these transcriptional dissimilarities, Ingenuity Pathway Analysis (IPA), Gene Set Variation Analysis (GSVA), and Gene Ontology (GO) tools were used to identify differentially expressed pathways and biological functions. IPA identified the most significant differences for the CCR6^+^DN versus CCR6^+^DP and Th17 versus CCR6^+^DP contrasts, and revealed similarities between CCR6^+^DN and Th17 (Fig. 2b). Among top modulated pathways, PPAR/RXR (previously associated with the negative regulation of Th17 polarization [35]), cyclin and cell cycle regulation, and apoptosis pathways were under represented in CCR6^+^DN versus CCR6^+^DP. In contrast, IL-6, phospholipase C (both involved in Th17 differentiation [29] CXCR4, thrombin, p38/ERK/MAPK (involved in cell activation/proliferation [36]) and Tec Kinase (involved in TCR signaling [37]) were over expressed in CCR6^+^DN versus CCR6^+^DP and in CCR6^+^DN versus Th17. Other pathways specifically up-regulated in CCR6^+^DN versus CCR6^+^DP and Th17 versus CCR6^+^DP included IL-2, Jak/STAT, PKC, sphingosine-1-phosphate (involved in cell migration [38]), NANOG (implicated in the maintenance of stem cells [39]), IL-3 (involved in lymphopoiesis [40]), PI3 K (involved in Th17 differentiation [10], April (involved in Th17 polarization [41], and NFAT (involved in IL-17A production [42]) (Fig. 2b).

GSVA identified canonical pathways (C2) and biological processes (C5) differentially expressed in CCR6^+^DN versus CCR6^+^DP and Th17 versus CCR6^+^DP (Additional file 3: S2A-B Figure). Among the top 50 differentially-expressed canonical pathways, the IL-23 pathway, which is key for Th17 polarization [43], was found to be enriched in CCR6^+^DN (Fig. 2c). Among the top 50 differentially-expressed biological processes, the expression of pathways such as lymphocyte differentiation, protease inhibitor and response to steroid hormone receptor were over represented in CCR6^+^DN (Fig. 2d).

GO identified differentially-expressed genes in CCR6^+^DN versus CCR6^+^DP (*p* < 0.05, cut-off 1.3-fold) involved in cell migration, chemotaxis, cell differentiation, and transcription. Transcripts associated with migration/chemotaxis into/outside lymph nodes, including CCR7, CXCR5, the CXCR5 ligand CXCL13, SELL, SIRP1, JAM3 and AIF1 were enriched in CCR6^+^DN, whereas adhesion molecules/chemokine receptors mediating homing into peripheral tissues such as integrin β7 (gut), CXCR3 (inflammatory sites), CCR2 and CCR4 (skin), and integrin β1 (cervix) were enriched in both Th17 and/or CCR6^+^DP (Additional file 3: S2C-D Figure). Well-established markers of Th17 (IL-8, IL-23R, SOSC3, TIM3, CD26/DPP4) and follicular helper T cells (Tfh) (CXCR5, ICOS) were enriched in CCR6^+^DN (Additional file 3: S2E Figure). Also, transcription factors related to the control of Th17 (STAT3, RORA) and Tfh (BLC6, ASCL2 [44, 45]) polarization/function were enriched in CCR6^+^DN (Additional file 3: S2E-F Figure). In addition, other transcripts enriched in CCR6^+^DN versus CCR6^+^DP include LEF1 and MYC, key markers of human stem-cells and long-lived Th17 cells [25], together with the anti-senescence marker TERC [46] (Additional file 2: Table S1). RT-PCR quantifications confirmed superior expression of STAT3, BCL6, LEF1 and TERC in CCR6^+^DN versus Th17 and CCR6^+^DP (Fig. 2e). These transcriptional analyses suggest that CCR6^+^DN, Th17 and CCR6^+^DP represent distinct stages of Th17 differentiation with specific migration potential, immunological functions, and transcriptional regulation. Among these subsets, CCR6^+^DN exhibit markers of early Th17 commitment, lymph-node tropism, follicular help, and self-renewal.

### CCR6^+^DN are a major source of IL-17F, IL-8 and IL-21

**Fig. 3:**
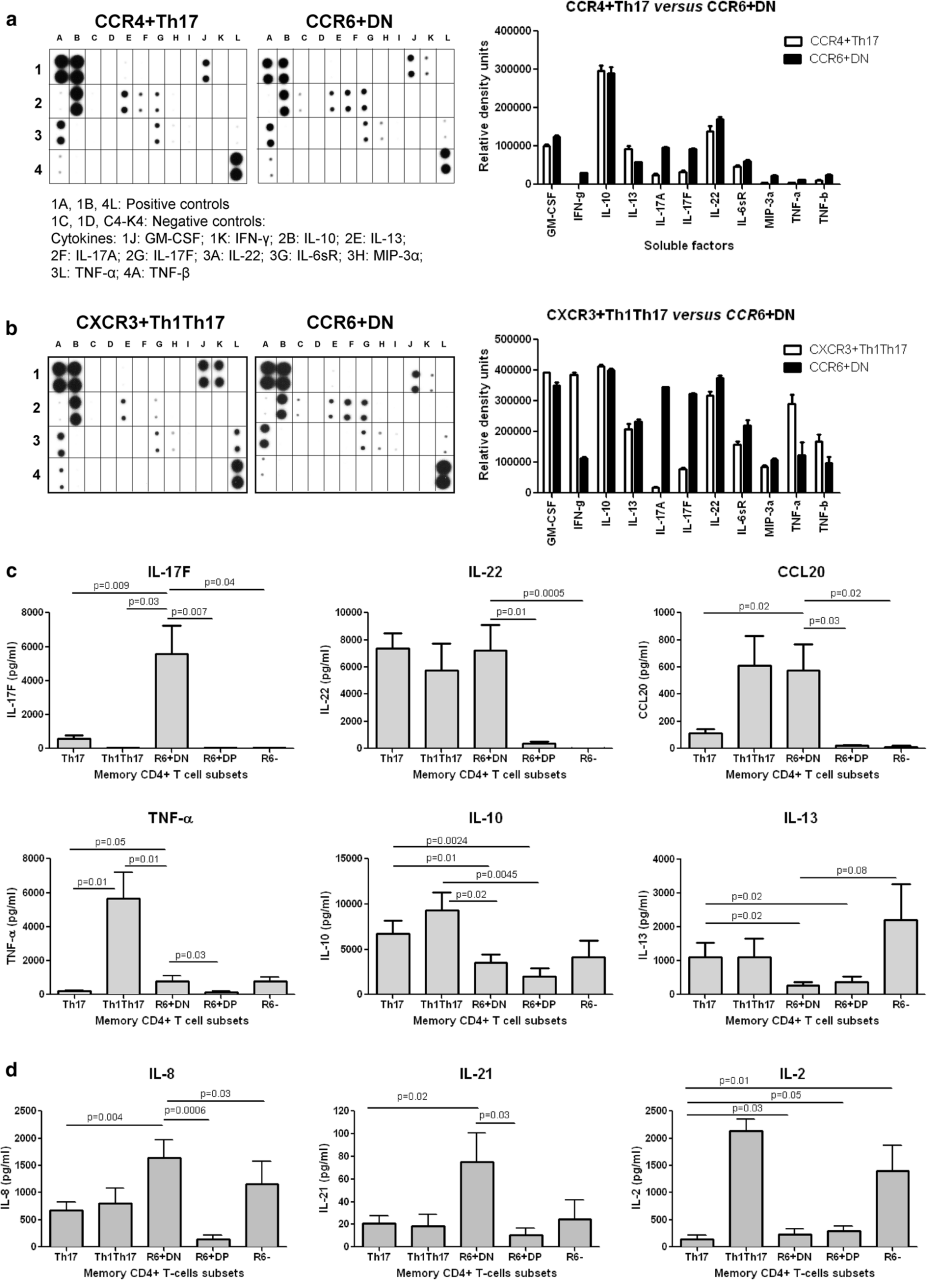
CCR6^+^DN cells are a major source of IL-17F, IL-8, and IL-21. Culture supernatants harvested from Th17, Th1Th17, and CCR6^+^DN (stimulated as in Fig. 1e) isolated from the peripheral blood of HIV-uninfected individuals were screened for the expression of 34 T-helper lineage-specific cytokines using the Human Th1/Th2/Th17 Antibody Array C series (RayBiotec). **a**, **b** Shown are results from one experiment with matched Th17 versus CCR6^+^DN and Th1Th17 versus CCR6^+^DN subsets: membrane blot (*left panels*) and relative density quantification (*right panels*). Results are representative of experiments performed with cells from two different donors: **c**, **d** Levels of IL-17F, IL-22, CCL20, IL-10, IL-13, TNF-α, IL-8, and IL-21 were quantified by ELISA. Shown are results on matched Th17, Th1Th17, CCR6^+^DN, CCR6^+^DP, and CCR6^−^ samples from different individuals (n = 3–7, mean ± SEM). Paired *t*-Test *p*-values are indicated in the *graphs*

A Cytokine Array was used to screen for the presence of 34 lineage-specific cytokines produced by CD3/CD28-activated CCR6^+^ subsets of HIV-uninfected individuals. CCR6^+^DN were distinguished from Th17 by increased IFN-γ, IL-17A, IL-17F, MIP-3α/CCL20 and TNF-α and decreased IL-13 (Fig. 3a), and from Th1Th17, by increased IL-17A and IL-17F, and decreased IFN-γ and TNF-α production (Fig. 3b). Levels of GM-CSF, IL-10 and IL-22 were similarly high in CCR6^+^DN, Th17 and Th1Th17 (Fig. 3a, b). ELISA quantifications demonstrated that CCR6^+^DN were the major source of IL-17F, produced IL-22 at levels similar to Th17 and Th1Th17, and CCL20 at levels higher and similar compared to Th17 and Th1Th17, respectively. In contrast, CCR6^+^DP produced low/undetectable levels of IL-17F, IL-22 and CCL20. Regarding the pro/anti-inflammatory profiles, Th1Th17 were major TNF-α and IL-10 producers [5, 15, 27], while CCR6^+^DN and CCR6^+^DP produced low levels of TNF-α and moderate levels of IL-10 and IL-13. Nevertheless, CCR6^+^DN produced slightly more TNF-α compared to Th17 or CCR6^+^DP (Fig. 3c). Although not detected in our cytokine screen, IL-8, IL-21, and IL-2 were quantified by ELISA. The highest levels of IL-8 and IL-21 were detected in CCR6^+^DN, while the highest levels of IL-2 were detected in Th1Th17 (Fig. 3d). Thus, in contrast to CCR6^+^DP which exhibit modest Th17 features, CCR6^+^DN are a major source of IL-17F, IL-8 and IL-21 and their pro-inflammatory profile is intermediate between that of Th17 and Th1Th17. Similar to CCR6^+^DP, CCR6^+^DN produce low IL-2 levels, a typical property of Th17 cells [15, 47].

### CCR6^+^DN proliferate and produce IL-17A in response to *C. albicans*

**Fig. 4:**
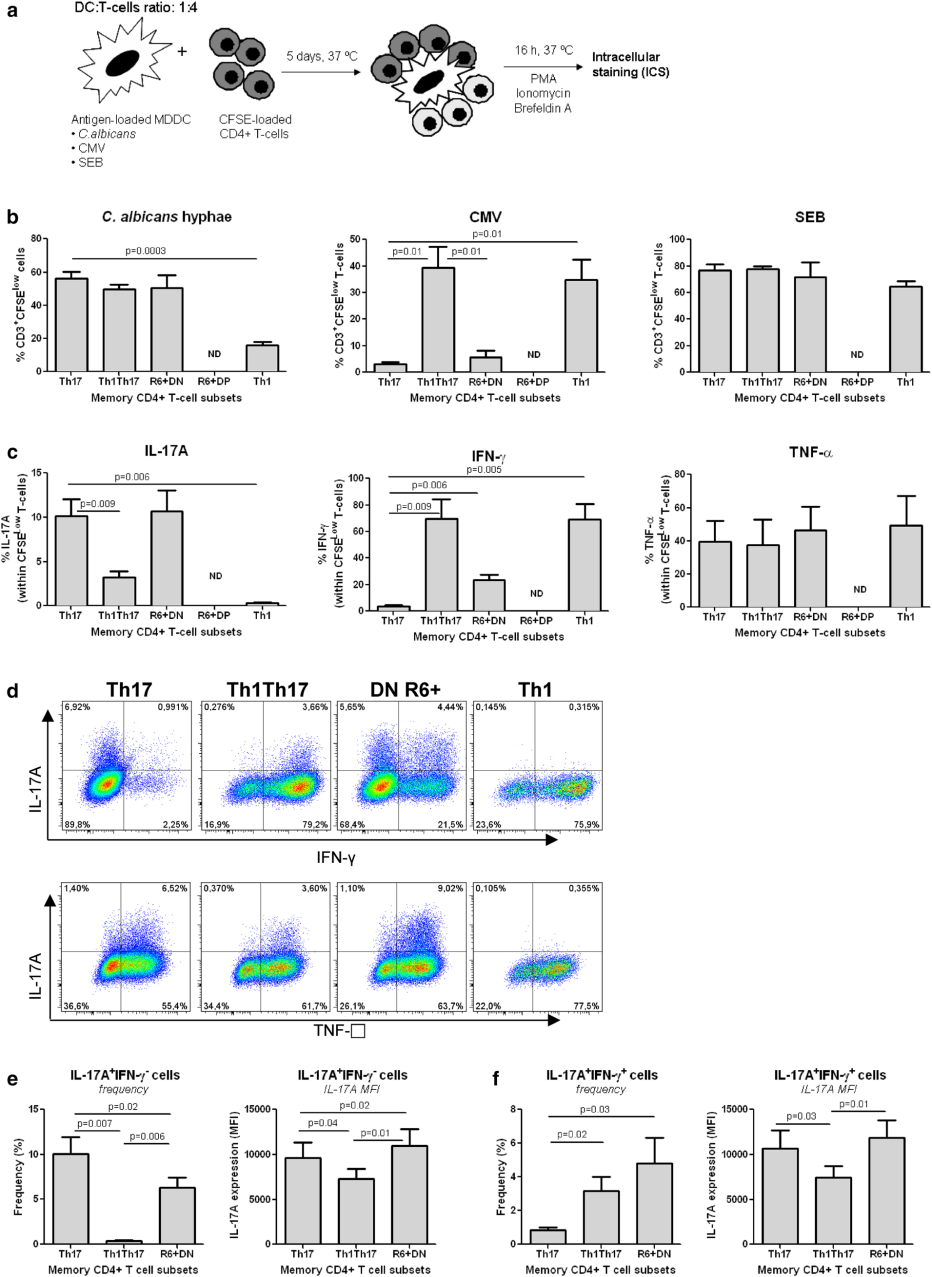
CCR6^+^DN proliferate in response to *C.albicans* but not CMV. **a** FACS-sorted T-cell subsets isolated from the peripheral blood of HIV-uninfected individuals were stained with CFSE, co-cultured with antigen-loaded autologous monocyte-derived dendritic cells (MDDC), and analyzed for their ability to proliferate (CFSE^low^) and produce cytokines. **b** Shown is the frequency of T-cells proliferating in response to *C. albicans* hyphae, CMV, or SEB at day 5 post co-culture. **c** Shown is the frequency of cytokine-expressing T-cells proliferating in response to *C. albicans* within each subset. **d**–**f** *C. albicans*-specific T-cells were further analyzed for the co-expression of IL-17A with IFN-γ or TNF-α. **d** Results are from one donor representative of results obtained with matched subsets from four different donors. **(e**–**f**) Shown is the frequency and MFI of *C. albicans*-specific T-cells expressing IL-17A either alone (IL-17A^+^IFN-γ^−^) (**e**) or in combination with IFN-γ (IL-17A^+^IFN-γ^+^) (**f**). **b**–**f** The positivity gates were defined based on FMO controls. **b**, **c** and **e**, **f** Shown are results (mean ± SEM) on matched samples from n = 4 different. Paired *t*-Test *p*-values are indicated in the *figures*. *ND* not determined

We further examined proliferation in response to typical Th17 (*C. albicans*) versus Th1 (CMV) antigens [5, 48] using a monocyte-derived dendritic cell (MDDC)-based antigen presentation assay (Fig. 4a). SEB was used to induce polyclonal proliferation. Sufficient numbers of CCR6^+^DP could not be sorted for these studies. Th17 and Th1Th17 proliferated in response to *C.albicans* at levels superior to Th1, while Th1Th17 and Th1 but not Th17 proliferated in response to CMV. Similar to Th17, CCR6^+^DN proliferated in response to *C. albicans* but not CMV. All subsets proliferated similarly well in response to SEB (Fig. 4b). In response to *C. albicans*, the frequency of IL-17A^+^ cells was similarly high in CCR6^+^DN and Th17, low in Th1Th17, and background in Th1 (Fig. 4c). The frequency of IFN-γ^+^ within *C. albicans*-specific CCR6^+^DN was lower compared to Th1Th17 and Th1 and higher compared to Th17. TNF-α was produced by all subsets at similarly high frequency.es. The frequency of IL-17A^+^ cells co-expressing IFN-γ was different between the three *C. albicans*-specific CCR6^+^ subsets, while the majority of IL-17A^+^ cells in all subsets co-expressed TNF-α (Fig. 4d). Notably, *C. albicans*-specific CCR6^+^DN included relatively high frequencies of IL-17A^+^IFN-γ^−^ and IL-17A^+^IFN-γ^+^ cells (Fig. 4e, f). Thus, CCR6^+^DN share antigenic specificity and cytokine profiles with the previously characterized Th17 and Th1Th17.

### CCR6^+^DN and CCR6^+^DP exhibit Th17-commitment and lineage plasticity

**Fig. 5:**
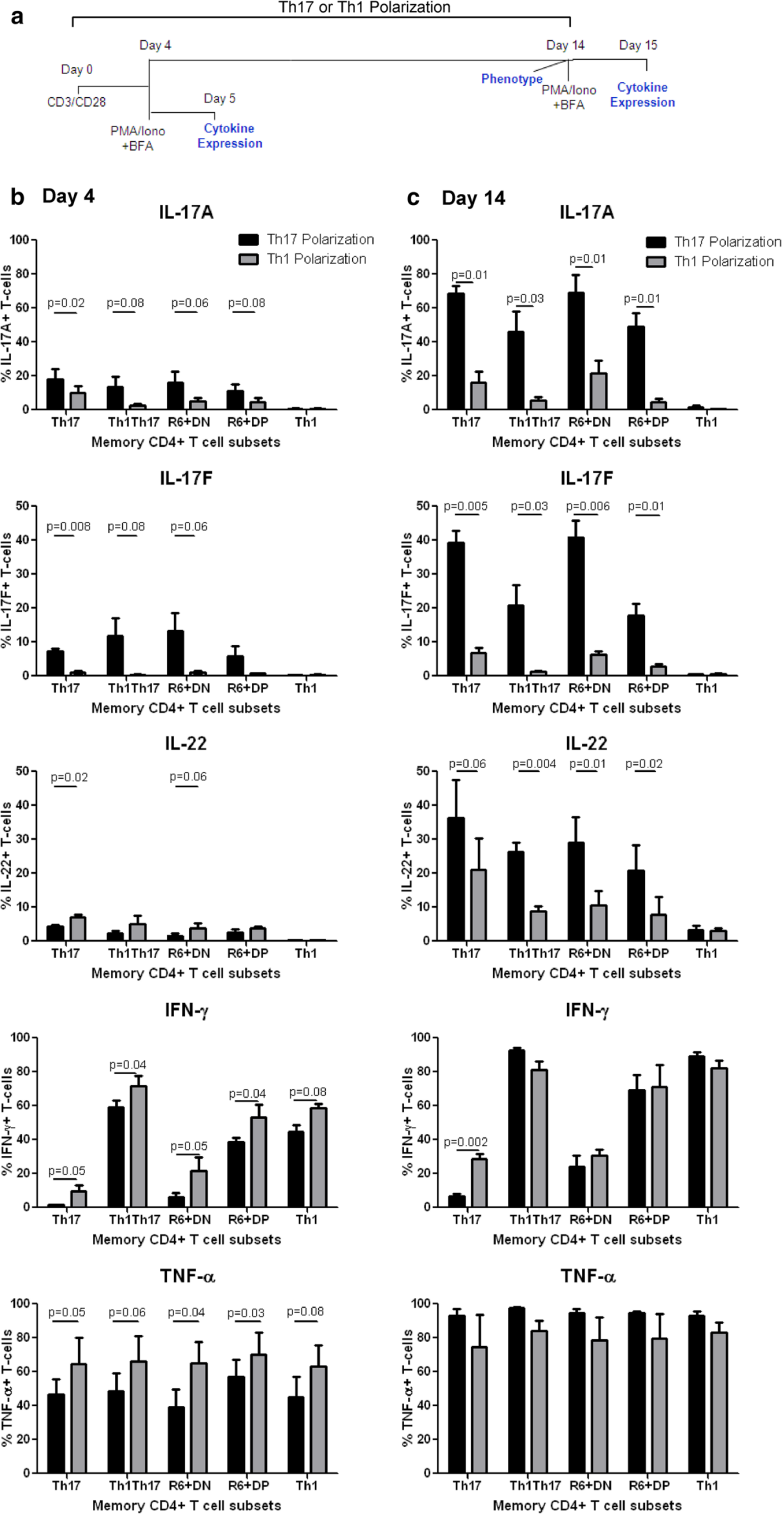
Lineage commitment *versus* plasticity of the four CM CCR6^+^ subsets in vitro. FACS-sorted CM (CD45RA^−^CCR7^+^) subsets isolated from the peripheral blood of HIV-uninfected individuals were analyzed for the expression of lineage-specific cytokines upon Th17/Th1-polarization in vitro. **a** CM subsets were stimulated via CD3/CD28 and cultured under Th17- (IL-1β, IL-6, and IL-23, and anti-IL-4 and anti-IFN-γ Abs) and Th1-polarizing conditions (IL-12 and anti-IL-4 Abs) for 4 and 14 days. Cells were stimulated with PMA and Ionomycin in the presence of Brefeldin A for 16 h. Intracellular staining was performed with cytokine-specific Abs. **b**, **c** Shown are statistical analyses of cytokines expressed by the distinct CM subsets cultured for 4 (**b**) or 14 days (**c**) under Th17- (*black bars*) and Th1-polarizing conditions (*grey bars*). Results (mean ± SEM) were generated with matches samples from n = 3 different donors. Paired t-Test *p*-values are indicated on the *figures* (Th17- versus Th1-polarization)

The Th17 polarization flexibility/plasticity is well documented [2, 29], with Th17 being able to down-regulate RORγt and acquire Th1 features in response to Th1-specific polarizing cues [6]. Therefore, we investigated the Th17-lineage commitment versus plasticity of the four CCR6^+^ subsets upon short (4 days) and long-term (14 days) culture under Th17- versus Th1-polarizing conditions (Fig. 5a). It is well-established that CM versus EM/TM are long-lived and acquire robust effector functions upon TCR triggering [32, 49]. Considering the superior production of Th17 cytokines by Th17-polarized CM versus EM/TM (Additional file 4: S3 Figure) as well as the preponderance of CM cells (Fig. 1d), subsequent experiments were performed using CM isolated from HIV-uninfected individuals. Under Th17-conditions, all four CCR6^+^ subsets produced IL-17A, IL-17F, IL-22 and TNF-α, while IFN-γ was highly expressed by Th1Th17, CCR6^+^DP and Th1 (Fig. 5b, c). The culture under Th17-conditions for 14 versus 4 days resulted in a remarkable increase in the proportion of cells producing Th17 effector cytokines. The culture under Th1- versus Th17-conditions resulted in a significant decrease in IL-17A, IL-17F and/or IL-22 expression by all CCR6^+^ subsets, with differences being more significant at day 14 versus 4. This was associated with increased IFN-γ and TNF-α expression by CCR6^+^ subsets at day 4 (Fig. 5b) and increased IFN-γ within Th17 at day 14 (Fig. 5c). As expected [29], Th1 did not acquire Th17-features under Th17-conditions (Fig. 5b, c). Changes in the poly-functionality of CM subsets cultured long-term under Th17 versus Th1-conditions (Additional file 5: S4 Figure) were further evaluated using SPICE [50] (Additional file 6: S5 Figure). Under Th17-conditions, CCR6^+^ subsets expressed 1–5 cytokines simultaneously. Th17 and CCR6^+^DN expressed IL-17A mainly in combination with TNF-α alone or with TNF-α and IL-17F and/or IL-22, but at relatively low frequency in combination with IFN-γ. Th1Th17 and CCR6^+^DP expressed IL-17A mainly in combination with IFN-γ and TNF-α, in the presence or absence of IL-17F or IL-22. The poly-functionality of CCR6^+^ subsets decreased under Th1- versus Th17-conditions and was associated with diminished expression of Th17-cytokines and expansion of IFN-γ^+^TNF-α^+^ cells, representative of a Th1 profile. Thus, similar to Th17 and Th1Th17, CCR6^+^DN and CCR6^+^DP amplified their Th17-features under Th17-conditions and acquired Th1-features under Th1-conditions. Nevertheless, fractions of Th17 and CCR6^+^DN preserved their Th17 features under Th1-conditions, indicative that stably-committed Th17 exist in humans.

### CCR6^+^DN acquire CCR4/CXCR3 expression in response to Th17- or Th1-polarizing signals

**Fig. 6:**
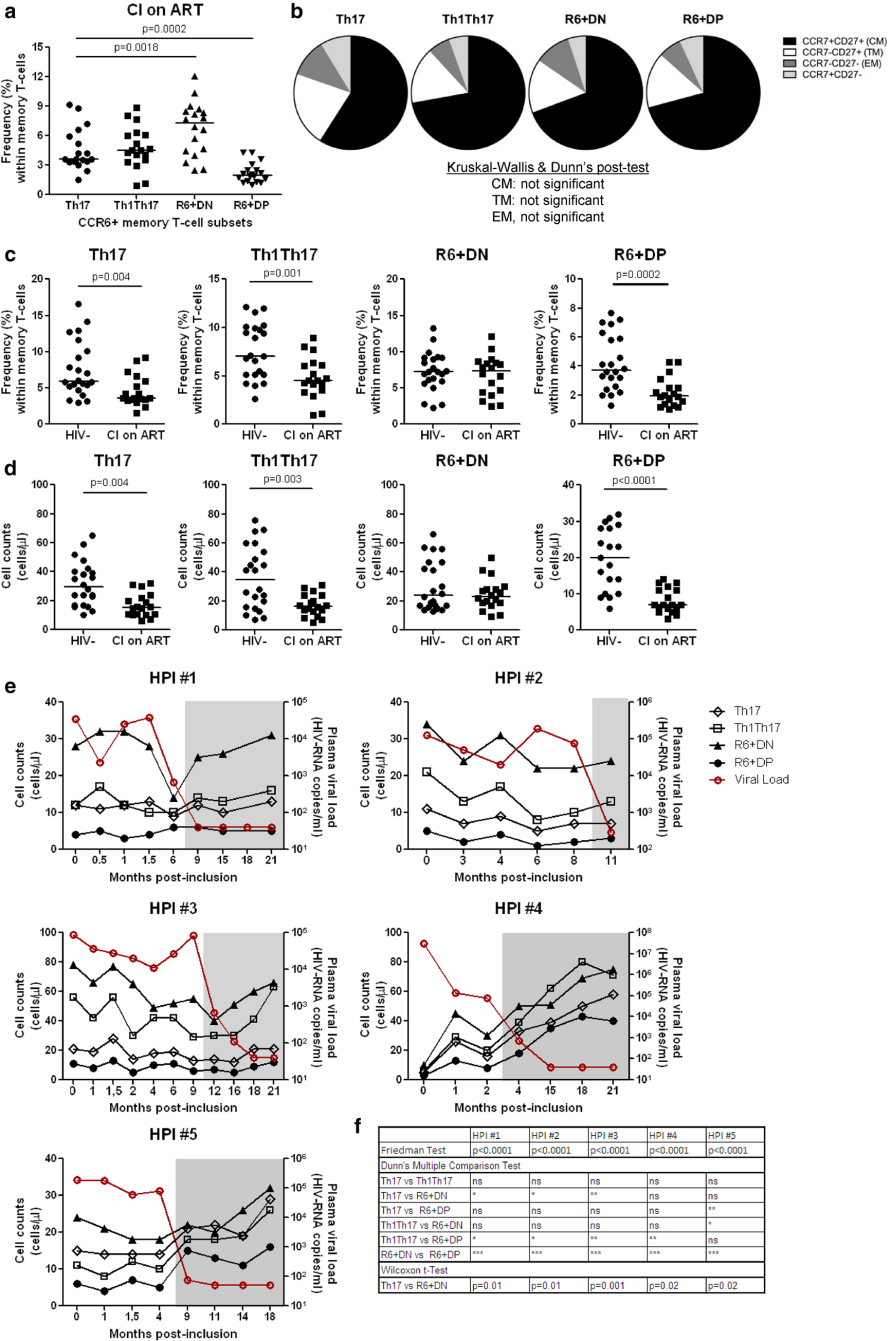
CCR6^+^DN distinguish from the other Th17-subsets by superior frequency/counts in CI on ART individuals. **a** PBMCs from CI on ART individuals (n = 20; Additional file 8: Table S2) were stained as in Fig. 1. Shown is the relative frequency of the four CCR6^+^ subsets in CI on ART individuals. Paired t-Test *p*-values are indicated on the *figures*. *Horizontal bars* indicate median values. **b** Shown are median frequencies of CM, TM, and EM within each CCR6^+^ subsets from CI on ART (n = 10). Shown is the frequency (**c**) and counts (**d**) of the four CCR6^+^ subsets in CI on ART versus uninfected individuals. Cell counts were calculated taking into account their frequency within the total CD4^+^ T-cell fraction. **e** The dynamics of CCR6^+^ subset counts were investigated longitudinally in n = 5 HIV-infected individuals from the Montreal HIV Primary infection cohort (Additional file 8: Table S3), in relationship with plasma viral load, before and after ART initiation (*grey*). **f** Shown are statistical analysis for differences in cell counts between the four CCR6^+^ subsets (n = 5 HIV-infected individuals) using Friedman test and the post-test Dunn’s multiple comparison and Wilcoxon *t* test

Finally, we investigated the stability/plasticity of CCR6, CCR4, and CXCR3 expression on CM Th17-subsets upon long-term exposure to Th17-/Th1-polarizing signals (Additional file 7: S6 Figure). Under Th17-conditions, CCR6 was significantly down-regulated compared to Day 0, while culture under Th1- *versus* Th17-conditions was associated with a further decrease in CCR6 expression, mainly on CXCR3^+^Th1Th17 (Additional file 7: S6A Figure). However, CCR6 expression was preserved at >70 and >50 % under Th17- and Th1-conditions, respectively (Additional file 7: S6A Figure). CCR4 expression was acquired by CXCR3^+^Th1Th17, CCR6^+^DN, and Th1 under Th17- or Th1-conditions (Additional file 7: S6B Figure). Notably, CCR6^+^DN cultured under Th17- or Th1-conditions acquired CCR4 at levels similar to CCR4^+^Th17 (Additional file 7: S6B Figure). In contrast, CCR4 expression decreased on CCR6^+^DP and CCR4^+^Th17 under Th17- or Th1-conditions (Additional file 7: S6B Figure). CXCR3 expression was decreased on CXCR3^+^Th1Th17, CCR6^+^DP, and Th1 under Th17- or Th1-conditions, but increased on CCR6^+^DN and CCR4^+^Th17 under Th17-, and even more dramatically Th1-conditions. However, CXCR3 levels remained inferior to those detected on CXCR3^+^Th1Th17 and CCR6^+^DP (Additional file 7: S6C Figure). Analysis of CCR6 co-expression with CCR4 or CXCR3 revealed that ~70 % of CCR4^+^Th17 and CXCR3^+^Th1Th17 maintained their original phenotype under Th17-conditions (Additional file 7: S6D-E Figure). Fractions of CXCR3^+^Th1Th17 (mean: 21 %) and CCR6^+^DN (mean: 48 %) acquired a CCR6^+^CCR4^+^ phenotype (Fig. 6d), while Th17 (mean: 29 %) and CCR6^+^DN (mean: 24 %) acquired a CCR6^+^CXCR3^+^ phenotype (Additional file 7: S6E Figure). The CCR6^+^CCR4^+^ and CCR6^+^CXCR3^+^ phenotype was dramatically reduced within CCR6^+^DP under Th17- or Th1-condititions (Additional file 7: S6D-E Figure), with CCR6^+^DP acquiring a CCR4^−^CXCR3^+^ but not CCR4^+^CXCR3^−^ phenotype. These results reveal the remarkable flexibility of CCR6/CCR4/CXCR3 expression on Th17-subsets upon TCR triggering and in relation with the cytokinic environment. Nevertheless, CCR6 expression was maintained on the majority of the four Th17-subsets, supporting the key role played by CCR6 in regulating tissue-specific homing of Th17-cells.

### Th17-polarized CCR6^+^DN and CCR6^+^DP are present in the blood of HIV-infected individuals

To determine whether CCR6^+^DN and CCR6^+^DP exist in the blood of HIV-infected individuals and are stably Th17-polarized, the four memory CCR6^+^ T-cell subsets as well as Th1 cells were identified and then sorted by flow cytometry (as in Additional file 1: S1 Figure) from three chronically infected aviremic ART-treated individuals (CI on ART) (Additional file 2: Table S2). The intracellular expression of IL-17A and IFN-γ was assessed upon long-term culture in vitro. Briefly, cells were stimulated via CD3/CD28 for 4 days and then cultured in the presence of IL-2 (Th0 polarizing conditions) for additional 9 days (Additional file 7: S6A Figure). Similar to HIV-uninfected donors, Th17 and CCR6^+^DN subsets from CI on ART individuals included a major fraction of IL-17A^+^IFN-γ^−^ cells, while Th1Th17, CCR6^+^DN, and CCR6^+^DP exhibited a similar frequency of IL-17A^+^IFN-γ^+^ cells (Additional file 7: S6B-C Figure). Of note, similar to Th1, Th1Th17 and CCR6^+^DP included a major fraction of IL-17A^−^IFN-γ^+^ cells (Additional file 7: S6B-C Figure). In conclusion, the two newly identified Th17-polarized subsets, CCR6^+^DN and CCR6^+^DP, exist in the peripheral blood of HIV-infected individuals and exhibit a Th17- and Th1Th17-polarization profile.

### CCR6^+^DN predominate in the blood of HIV-infected individuals during ART

Circulating Th17 and Th1Th17 are depleted upon HIV-infection and their frequency is not restored with viral-suppressive ART [15, 16]. Thus, we investigated alterations in the frequencies of CCR6^+^DN and CCR6^+^DP, relative to Th17 and Th17Th17, during HIV infection. In contrast to uninfected controls (Fig. 1b), the frequency of CCR6^+^DN in CI on ART individuals (Additional file 2: Table S2) was superior to that of Th17 and Th1Th17, while CCR6^+^DP were the least preponderant subset (Fig. 6a). As in uninfected controls (Fig. 1d), the four CCR6^+^ subsets from CI on ART individuals were enriched in cells with a CM phenotype (Fig. 6b). Similar to Th17 and Th1Th17 [15], the frequency of memory CCR6^+^DP was significantly reduced in CI on ART versus uninfected individuals (Fig. 6c). In contrast, no significant differences were observed in the frequency of CCR6^+^DN in CI on ART versus uninfected individuals. Similar observations were made when absolute cell counts were compared (Fig. 6d), indicative that CCR6^+^DN are preserved during HIV infection.

To further explore the preservation of CCR6^+^DN during HIV infection, we performed longitudinal studies in HIV-infected individuals enrolled in the Montreal HIV Primary Infection (HPI) cohort. The dynamics of the four CCR6^+^ subsets were studied in relationship with plasma viral load at different time-points post-infection, before and after ART initiation (Fig. 6e, f). Of note, in all five HIV^+^ individuals, CCR6^+^DN were the most predominant CCR6^+^ subset before and after ART initiation. ART initiation during the first year of infection efficiently decreased plasma viral load to undetectable levels in all individuals (Fig. 6e) and increased CD4 counts (Additional file 8: Table S3). We observed a tendency for increased Th17, Th1Th17 and CCR6^+^DN counts following treatment (Fig. 6e), indicative of the positive effects of ART in increasing Th17 levels during early chronic infection. The counts of CCR6^+^DP were increased in 4/5 individuals following treatment but remained stable in one individual. Interestingly, the counts of CCR6^+^DN were superior at all time-points in 4/5 and 5/5 individuals, respectively, when compared to Th17 and CCR6^+^DP. In contrast, CCR6^+^DP counts were the lowest in all patients before/after ART initiation. These results demonstrate that CCR6^+^DP but not CCR6^+^DN are depleted in CI on ART individuals.

### CCR6^+^DN are enriched in the lymph nodes of HIV-infected individuals on ART

**Fig. 7:**
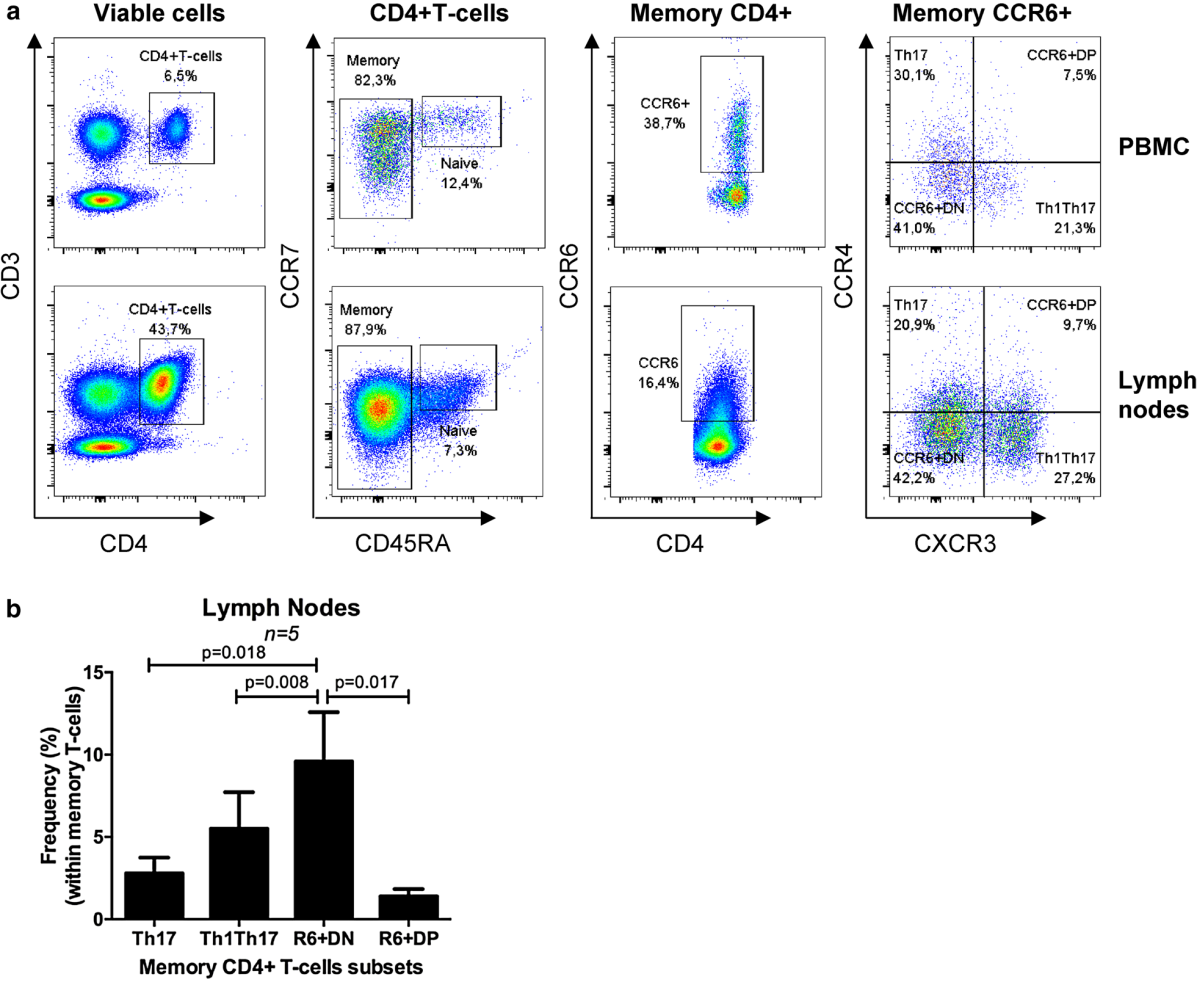
CCR6^+^DN are predominant in lymph nodes of HIV-infected individuals receiving ART. **a**, **b** Matched PBMCs and inguinal lymph node cells from three CI on ART individuals (CI 36, CI 37, CI 38; Additional file 8: S2 Table) were stained with a cocktail of fluorochrome-conjugated CD3, CD4, CD45RA, CCR4, CXCR3, CCR6, and CCR7 Abs. A viability staining was used to exclude dead cells. Viable memory CD4^+^ T-cells (CD3^+^CD4^+^CD45RA^−^) expressing CCR6 were analyzed for their differential expression of CCR4 and CXCR3. The four CCR6^+^ subsets including Th17, CCR6^+^DP, CCR6^+^DN, and Th1Th17 were identified in both PBMCs and cells from lymph nodes. **a** Shown is the phenotype of PBMCs (*upper panels*) and lymph node cells (*lower panels*) in one representative donor. **b** Shown are statistical analysis of the frequency of CCR6^+^ subsets in the lymph node (n = 3). Paired t-Test *p*-values are indicated on the *figures*

Blood CCR6^+^DN express Tfh markers as demonstrated by transcriptional profiling (Fig. 2) and IL-21 quantification (Fig. 3D). Tfh are localized in the B cell follicles of secondary lymphoid organs and are major targets for HIV infection [51, 52]. Access to inguinal lymph nodes from three HIV-infected individuals on ART (Additional file 8: Table S2; CI 36, CI 37, CI 38) allowed us to reveal that CCR6^+^DN represent the most frequent CCR6^+^ T-cell subset in the lymph nodes (Fig. 7). These results suggest that peripheral blood CCR6^+^DN recirculate preferentially through lymph nodes and may include a fraction of Tfh.

### CCR6^+^DN are permissive to viral infection in vitro and in HIV-infected individuals

**Fig. 8:**
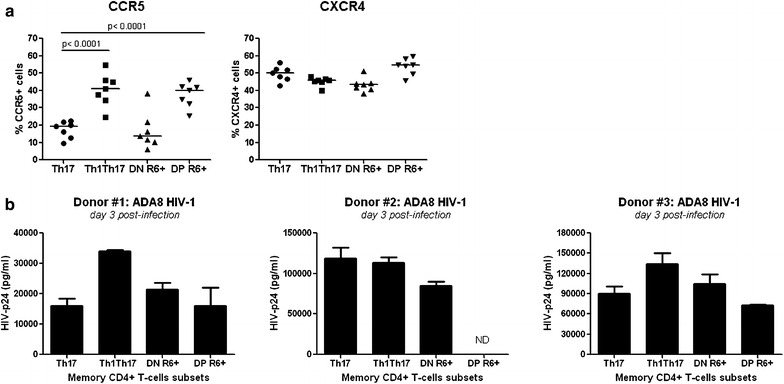
CCR6^+^DN and CCR6^+^DP subsets are permissive to HIV infection in vitro. **a** PBMCs from healthy individuals were stained with a cocktail of fluorochrome-conjugated CD3, CD4, CD45RA, CCR4, CXCR3, CCR6, and CCR5 or CXCR4 Abs. The gating strategy for the identification of distinct CCR6^+^ and CCR6^−^ T-cell subsets was designed as in Fig. 1a. The frequency of cells expressing CCR5 (*left panel*) and CXCR4 (*right panel*) was analyzed within the Th17, Th1Th17, CCR6^+^DN and CCR6^+^DP subsets. Paired t-Test *p*-values are indicated on the figures. Horizontal bars indicate median values. **b** Memory CCR6^+^ subsets from three HIV-uninfected subjects were sorted and stimulated via CD3/CD28 for 4 days, as in Fig. 1e. Cells were exposed to a highly infectious R5 strain HIV-ADA8. Levels of HIV-p24 were quantified by ELISA in cell supernatants at day 3 posy-infection

We and others previously demonstrated that Th17 and Th1Th17 cells are permissive to HIV infection [15, 16]. Thus, CCR6^+^DN and CCR6^+^DP were compared to Th17 and Th1Th17 in terms of HIV permissiveness in vitro and ability to carry proviral DNA in HIV-infected individuals. The HIV co-receptor CCR5 was expressed at low levels on CCR6^+^DN, similar to Th17, and high levels on CCR6^+^DP, similar to Th1Th17. CXCR4 expression was similarly high on all subsets (Fig. 7a). Exposure to replication-competent R5 HIV strain ADA8 demonstrated that CCR6^+^DN and CCR6^+^DP, similar to Th17 and Th1Th17, are permissive to HIV infection in vitro (Fig. 7b). Further, Th17, Th1Th17, CCR6^+^DN and/or CCR6^+^DP were isolated from three recently-infected viremics untreated individuals (RI) (Additional file 8: Table S2) for integrated HIV-DNA quantification. Results in Additional file 9: S7 Figure demonstrate that similar to Th17 and Th1Th17, CCR6^+^DN carry integrated HIV-DNA in all three donors tested, at relative levels varying between donors but superior to levels in naive T-cells. Thus, CCR6^+^DN are permissive to HIV infection in vitro and in vivo (Fig. 8).

### CCR6^+^DN carry replication-competent integrated HIV-DNA in CI on ART individuals

**Fig. 9:**
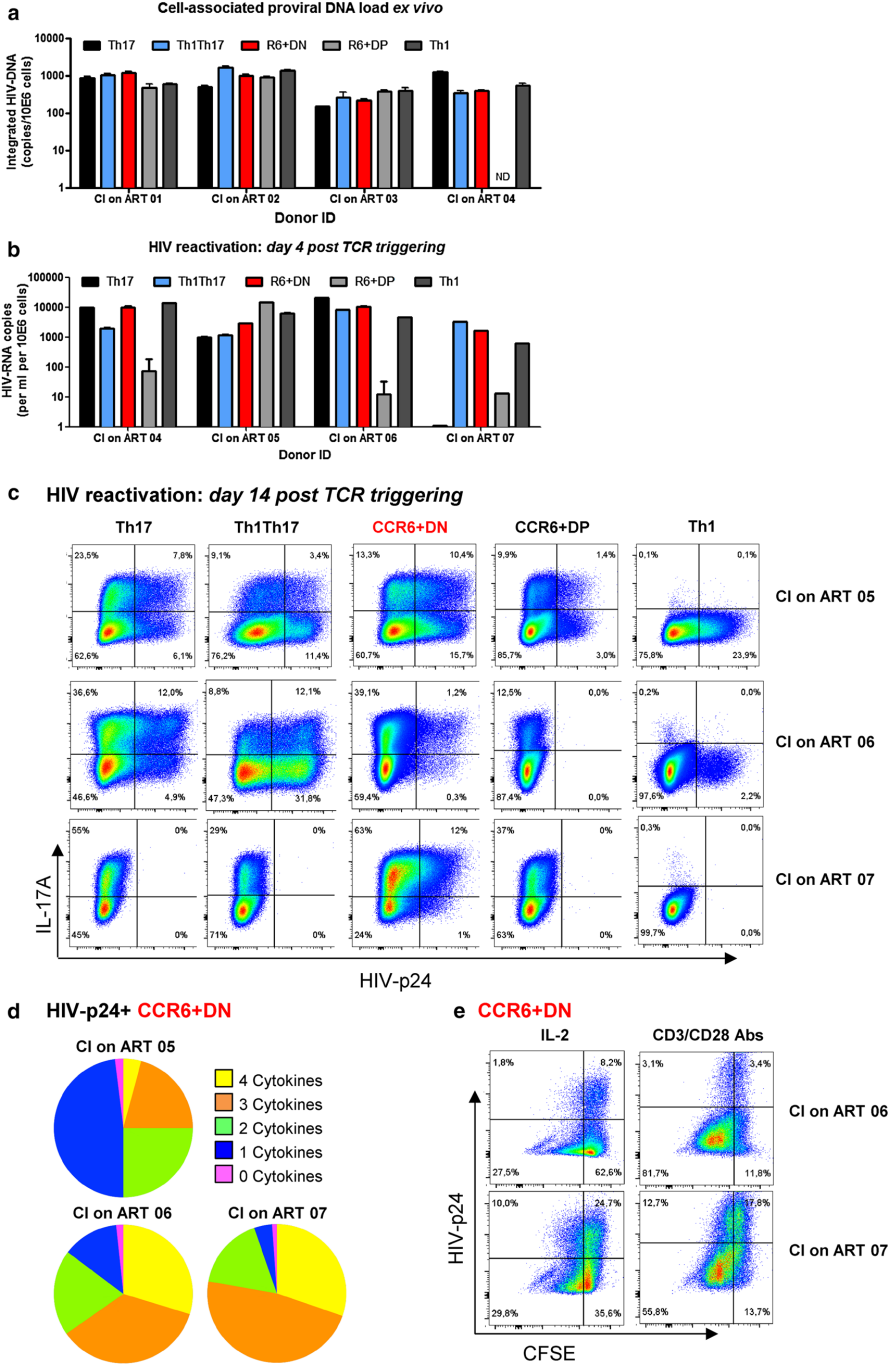
CCR6^+^DN carry replication competent HIV-DNA. **a**, **b** The four memory CCR6^+^ subsets as well as Th1 cells from PBMCs of chronically infected receiving viral suppressive ART (CI on ART) individuals were sorted by FACS. **a** Levels of integrated HIV-DNA were quantified by nested real-time PCR in sorted cells ex vivo (mean ± SD of triplicate wells; n = 4 CI on ART individuals). **b**, **c** FACS-sorted memory subsets were stimulated via CD3/CD28 and cultured as described in S6A Figure legend for up to 14 days. **b** HIV-RNA levels were quantified by real-time RT-PCR in culture supernatant of cells stimulated via CD3/CD28 for 4 days (n = 4 CI on ART individuals). **c**, **d** At day 13, cells were stimulated with PMA and Ionomycin in the presence of Brefeldin A for 6 h. Intracellular staining was performed with cytokine-specific Abs and HIV-p24. **c** Shown are flow cytometry dot plots illustrating the co-expression of IL-17A and HIV-p24 (n = 3). **d** Shown are pie charts representations generated with the SPICE software illustrating the poly-functional profile of HIV-p24^+^ CCR6^+^DN cells; all possible combinations of one (*blue*), two (*green*), three (*orange*) and four (*yellow*) or no (*purple*) cytokines are depicted (n = 3 CI on ART individuals). **e** At day 13, CCR6^+^DN subsets were stained with CFSE and cultured for 5 additional days in the presence of either IL-2 (5 ng/ml) or CD3/CD28 (1 µg/ml). Cells expressing or not intracellular HIV-p24 were then analyzed for their ability to proliferate (CFSE^low^)

Recent evidence from our group (Gosselin et al, unpublished observations) and others [26], support the contribution of long-lived Th17 cells to HIV reservoir persistence under ART. Thus, we sought to determine whether CCR6^+^DN harbor replication-competent HIV reservoirs in HIV-infected individuals with undetectable viral load under ART (Additional file 8: Table S2). The four CCR6^+^ T-cell subsets as well as Th1 cells were sorted by flow cytometry as in S1 Figure. Similar to the other cell subsets, CCR6^+^DN harbored integrated HIV-DNA ex vivo (Fig. 9a) and produced measurable levels of viral particles after stimulation via CD3/CD28 for 4 days (Fig. 9b), indicating that these integrated genomes can be induced to produce HIV virions. To further determine the replication competency of HIV virions, we performed a modified viral outgrowth assay [53] that consisted in the culture of CD3/CD28-activated cells for thirteen days in the presence of IL-2 (as in Additional file 1: S6A Figure). Results in Fig. 9c demonstrate intracellular expression of HIV-p24 in CCR6^+^DN from 3/3 CI on ART individuals, as well as the co-expression of HIV-p24 with IL-17A. The HIV-24 expression was also observed at various frequencies in Th17, Th1Th17 and Th1 cells in 2/3 CI on ART individuals Fig. 9c. Interestingly, HIV-p24 was detected in CCR6^+^DP only 1/3 CI on ART individuals. Of note, in preliminary experiments, intracellular HIV-p24 was detected when cells were cultured in the absence but not the presence of antiretroviral drugs (ARVs: AZT (180 nM), Efavirens (100 nM), Raltegravir (200 nM)), suggesting that cell-to-cell HIV spreading occurred in culture (data not shown). Noteworthy, the HIV-p24^+^ CCR6^+^DN cells probed to be highly poly-functional in terms of IL-17A, IL-22, IFN-γ and TNF-α expression (Fig. 9d; Additional file 10: S8 Figure). Finally, we observed that a fraction of the HIV-p24^+^ CCR6^+^DN cells were able to proliferate when cultured for additional 5 days in the presence of IL-2 or CD3/CD28 Abs (Fig. 9e). Together these results support the contribution of CCR6^+^DN cells to HIV persistence during ART.

## Discussion

The concept of Th17 pathogenicity is well defined in the context of autoimmunity [18, 19, 21]. During HIV-1 infection, Th17 cells may be considered pathogenic because they are permissive to HIV-1 and subsequently depleted [15, 16]. Alternatively, long-lived Th17 cells [25, 28, 29] may carry integrated HIV-DNA and contribute to viral reservoir persistence under ART (Gosselin et al, unpublished observations) [26]. In this manuscript we used a systems biology approach to characterize the heterogeneity of memory subsets expressing the Th17 marker CCR6 at homeostasis and during HIV-1 infection. We reveal the existence of two new subsets of Th17-polarized CCR6^+^ T-cells, CCR6^+^DN (CCR4^−^CXCR3^−^) and CCR6^+^DP (CCR4^+^CXCR3^+^), that share functional characteristics with the previously characterized Th17 and Th1Th17 [5, 15, 16] in terms of lineage-specific cytokines, antigenic specificity, lineage specification versus plasticity and HIV permissiveness. Genome-wide transcriptional profiling revealed a unique molecular signature in CCR6^+^DN, suggesting they represent an early stage of Th17 differentiation. In contrast to the other Th17-polarized subsets, the CCR6^+^DN was the most predominant in the blood and the lymph nodes of HIV-infected subjects with undetectable plasma viral load under ART. Finally, we demonstrated that CCR6^+^DN carry replication competent HIV reservoirs in ART-treated individuals. Our results support the concept that long-lived Th17 cells, *i.e.,* CCR6^+^DN, contribute to HIV persistence during ART.

Similar to Th17 cells, CCR6^+^DN were a major source of IL-17A, while CCR6^+^DP and Th1Th17 cells produced low IL-17A levels. Nevertheless, CCR6^+^DN and CCR6^+^DP expressed both typical Th17 transcripts (*i.e.,* IL-26, CCL20, IL-22, IL-23R, RORC, IL-17F, RORA, KLRB1/CD161, IL6R [3]) and potentially new Th17 markers (CXCR6, PTPN13, MAP3K4, S100A4, CD96, Ly9, KLF2, LGMN, TNFRSF25, ANTXR2, and CTSH). PTPN13, a phosphatase associated with Fas [54], was previously identified as Th17-specific [21, 55] and may contribute to the regulation of cell activation/apoptosis. ARNTL, a component of the circadian clock machinery, was proved to regulate RORγt expression and Th17 development in mice [56]. Finally, the expression of ANTXR2 is consistent with recent findings that Th17 cells govern adaptive immune responses against *Bacillus anthracis* [57].

Despite several transcriptional similarities, CCR6^+^DN, CCR6^+^DP and Th17 expressed distinct molecular signatures linked to specific pathways and biological processes. CCR6^+^DN were enriched in molecules associated with lymph node chemotaxis (CCR7, CXCR5, CXCL13, SELL, SIRP1, JAM3, AIF1). In contrast, Th17 and CCR6^+^DP expressed molecules related to homing into peripheral tissues including the gut (integrin β7 or CXCR3), skin (CCR2, CCR4) and cervix (integrin β1). Several signaling pathways involved in Th17 differentiation, including IL-6, phospholipase C, April and NFAT, were upregulated in CCR6^+^DN versus Th17 and/or CCR6^+^DP. CCR6^+^DN expressed higher levels of STAT3 mRNA and were enriched in other Th17-associated markers including IL-17F, RORA [58], IL-23R [43], SOSC3 [59], TIM3 [60] and CD26/DPP4 [61]. STAT3 is one of the first transcription factors to be up-regulated within the first hours of Th17 polarization [62]. Cell fate mapping experiments demonstrated IL-17F is expressed during early stages of Th17 differentiation [33]. Further, CCR6^+^DN were found to express a stem-cell-like molecular signature including RORA, STAT3, LEF1, MYC and TERC [25, 46, 63, 64] as well as the NANOG signaling pathway, important for the maintenance of stem cells [39]. LEF1 and MYC characterize long-lived Th17 cells with self-renewal properties [25]. TERC is linked to telomerase activity, which is up-regulated in stem cells, cancer cells and proliferating lymphocytes [46]. In contrast, CCR6^+^DP [56] together with Th17 expressed high levels of LMNA, a senescence marker [34]. These findings feed the idea that CCR6^+^DN versus Th17 and CCR6^+^DP represent a less advanced stage of Th17 differentiation with superior survival potential.

In addition to IL-17F, CCR6^+^DN are distinguished from the other CCR6^+^ subsets by their relatively high production of IL-8 and IL-21. IL-8 is critical for neutrophil recruitment at inflammatory sites [65], indicative of superior effector functions for CCR6^+^DN. IL-21 is the hallmark cytokine for follicular helper T cells (Tfh) [44]. IL-21 up-regulates expression of the Tfh-specific transcription factor BCL6 and the chemokine receptor CXCR5 [66]. Consistently, CCR6^+^DN express the highest levels of BCL6 and CXCR5 mRNA, and are also enriched in ASCL2 mRNA, another Tfh-specific transcription factor [45]. Thus, CCR6^+^DN cells share Tfh features. This is in line with a proposed differentiation model in which Tfh development occurs subsequent to Th1, Th2 or Th17 polarization [44]. Similar to Th17 but in contrast to Th1Th17 [15], CCR6^+^DN and CCR6^+^DP produce low/undetectable levels of IL-2, a cytokine known to promote cell survival [67]. IL-21 acts as a survival factor for Th17 cells [68]. IL-21 supplementation leads to the restoration of IL-17A-producing cells in SIV-infected macaques [68]. Therefore, IL-21 produced by CCR6^+^DN may act in autocrine manner to support superior survival of these cells and compensate for their limited IL-2 expression.

Th17 cells mediate immunity against pathogens localized at barrier surfaces including *C. albicans* [3, 5]. Here, we reveal that CCR6^+^DN, similar to Th17, proliferate and produce IL-17A in response to *C.albicans* but not CMV. We also observed proliferation of Th1Th17 and Th1 in response to *C. albicans* and CMV. These results are consistent with recent studies from *Becattini* et al. in which Th17, Th1Th17 and Th1 share *C.albicans* clonotypes, while expressing distinct effector cytokines [27]. Clonotype sharing between Th17 subsets is consistent with previous reports on the acquisition of Th1 features by Th17 cells under inflammatory conditions [29]. Whether CCR6^+^DN and Th17 share *C.albicans* clonotypes remains to be investigated. The antigenic specificity of CCR6^+^DP was not tested in our study. However, Becattini et al. identified a population similar to CCR6^+^DP that shared with Th1Th17 *M. tuberculosis* clonotypes [27], thus suggesting a potential differentiation relationship between CCR6^+^DP and Th1Th17.

In contrast to CCR6^−^, CCR6^+^ T-cells are prone to express Th17 functions [10]. Consistently, the frequency of cells expressing IL-17A, IL-17F and IL-22 was dramatically increased in all CCR6^+^ subsets upon long-term exposure to Th17-polarizing signals. Notably, CCR6^+^DN and Th17 were distinguished from CCR6^+^DP and Th1Th17 by their superior IL-17A/IL-17F and reduced IFN-γ expression. Th17 cells are known to be extremely plastic and can dedifferentiate into other lineages [29]. As such, all Th17 subsets diminished expression of Th17 cytokines and enhanced IFN-γ expression in response to Th1-polarizing signals. This is consistent with other studies in mice and humans demonstrating suppression of Th17 effector cytokines upon IL-12 exposure [6, 69]. Our results reveal that CCR6^+^DN and CCR6^+^DP, together with Th17 and Th1Th17, are four distinct Th17-commited subsets that exhibit lineage plasticity. Noteworthy, a fraction of CCR6^+^DN and Th17 preserved production of Th17 cytokines under Th1 conditions, providing evidence that stably-committed Th17 cells exist in humans, as previously predicted [2]. Our results also confirm the stability of the Th1 transcriptional program [29].

The chemokine receptors CCR6, CCR4, and CXCR3 mediate T-cell recruitment into gut-associated lymphoid tissues [7, 70], skin [71], and various other inflammatory sites [72]. We observed that the expression of these receptors represents stable signatures for a fraction of CM Th17-subsets upon exposure to Th17/Th1-polarization signals. In contrast to CCR4 and CXCR3, the expression of CCR6 was particularly stable under Th17-polarizing conditions, consistent with the identification of stable epigenetic mechanisms involved in the regulation of CCR6 expression [73]. CCR6 expression was mainly decreased upon exposure to Th1-polarizing cytokines, consistent with the reported ability of IL-12 to down-regulate CCR6 [74]. The expression of CCR4 and CXCR3 was stable on >70 % Th17 and >80 % Th1Th17, respectively, regardless of the polarization conditions. Interestingly, CCR6^+^DN cultured under Th17- or Th1-conditions acquired a “classical” Th17-phenotype (CCR6^+^CCR4^+^), while CXCR3, a marker for Th1Th17 and Th1-cells [5, 15], was similarly acquired by CCR6^+^DN and Th17 under Th17-/Th1-conditions. It is reported that Th1Th17-cells derive from Th17-cells that acquired Th1-features under inflammatory conditions [75–77]. Our results demonstrate that CCR6^+^DN acquire Th17 and/or Th1Th17 phenotypic features. In contrast to CCR6^+^DN, a significant fraction of CCR6^+^DP lost CCR4 and/or CXCR3, regardless of polarization conditions, and acquired a Th1Th17 but not Th17 phenotype. All these findings reveal flexibility in the expression of CCR6, CCR4 and/or CXCR3 by these Th17-subsets, flexibility that may be relevant for their differential recruitment into various tissues. This flexibility suggests a developmental relationship between these four Th17-subsets via molecular mechanisms that remain to be further investigated.

Of particular importance, we demonstrate that the four CCR6^+^ T-cell subsets exist in the peripheral blood of HIV-infected individuals and exhibit a Th17-polarization profile similar to subsets from uninfected individuals. The CCR6^+^DN were the most preponderant among Th17 subsets in the peripheral blood of ART-treated individuals as their frequency/counts were preserved at levels similar to uninfected individuals. Furthermore, in a longitudinal follow-up in five HIV-infected individuals, CCR6^+^DN proved to be the most predominant CCR6^+^ subset before/after ART initiation. This raised the possibility that CCR6^+^DN are resistant to infection.

In contrast to the above prediction, we demonstrated that all four Th17 subsets, including CCR6^+^DN, were permissive to HIV infection in vitro and in vivo. CCR6^+^DN and CCR6^+^DP expressed the HIV coreceptors CCR5 and CXCR4 at levels similar to Th17 and Th1Th17, respectively. Accordingly, all Th17 subsets were permissive to HIV in vitro. Furthermore, CCR6^+^DN, Th17 and Th1Th17 harbored proviral HIV-DNA in RI individuals, indicative of their HIV permissiveness in vivo. Of particular interest, all four CCR6^+^ subsets were found to carry relatively high levels of integrated HIV-DNA in virologically suppressed ART-treated individuals. We further demonstrated that viral transcription and production, reflected by the detection of HIV-RNA in cell culture supernatants, occurred in all four Th17 subsets, including CCR6^+^DN cells. Finally, we demonstrate active viral replication in CCR6^+^DN cells cultured for 13 days under Th0 polarizing conditions in vitro. Of note, HIV-p24^+^ CCR6^+^DN cells from CI on ART individuals co-expressed IL-17A and were highly poly-functional, and a fraction of them were able to proliferate in response to repetitive IL-2 stimulation or TCR triggering in vitro. These results support the contribution of CCR6^+^DN cells to HIV reservoir persistence under ART. This is consistent with a model in which integrative infection is compatible with survival [78] and CCR6 triggering via CCL20 promotes HIV latency [79] and suggests that permissiveness to HIV integration compatible with survival represents a new previously unrecognized feature of pathogenic Th17 cells during HIV infection. Future functional validations on the host cell machinery components used by HIV to insure its persistence in specific Th17 subsets such as CCR6^+^DN cells are important for the design of new targeted “*shock and kill*” strategies. Alternatively, other strategies may involve the interference with the CCL20-CCR6 interaction using small molecule inhibitors that may limit CCL20-mediated establishment of HIV latency in CCR6^+^ T-cells, consistent with the model proposed by Lewin et al. [79]. The fact that CCR6^+^DN exhibit CM features and are located in the lymph nodes emphasized the complexity/challenge of HIV eradication.

Our results that CCR6^+^DN express Tfh markers are in line with a recent study demonstrating the restoration of CCR6^+^ T cells expressing a Tfh signature (CCR7^high^CXCR5^high^PD-1^high^) under ART [81]. CD4^+^ T cells with CM features constitute the main reservoir of latent HIV [82]. Indeed, we demonstrate that CCR6^+^DN are enriched in cells with CM phenotype and they are the most predominant CCR6^+^ T-cell subset in the inguinal lymph nodes of three ART-treated individuals. Future studies are required to determine whether CCR6^+^DN from lymph nodes exhibit Tfh features and carry replication-competent HIV reservoirs.

## Conclusions

**Fig. 10:**
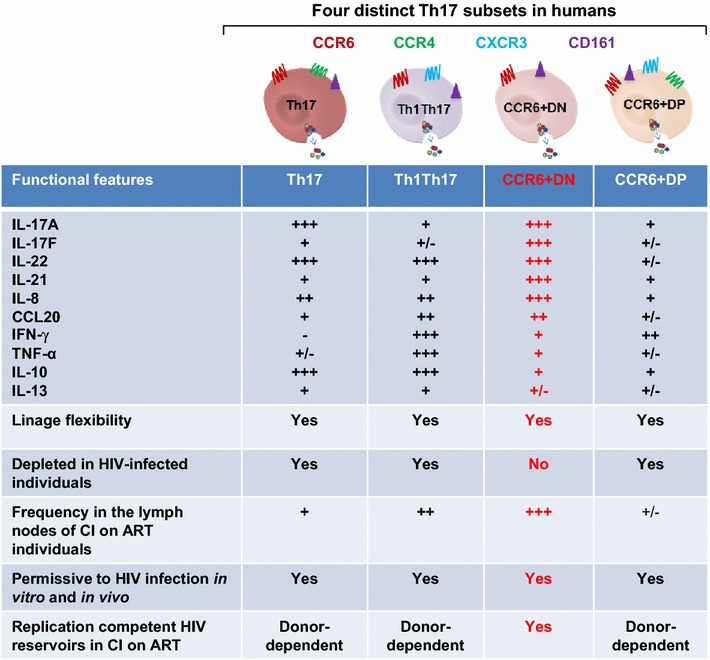
New insights into the heterogeneity of human Th17 cells at homeostasis and during ART-controlled HIV-1 infection. In this work we identified two new subsets of CCR6^+^ T-cells, CCR6^+^DN/CCR4^−^CXCR3^−^ and CCR6^+^DP/CCR4^+^CXCR3^+^, that share Th17 features with the previously described Th17/CCR4^+^CXCR3^−^ and Th1Th17/CCR4^−^CXCR3^+^ [5]. Despite these similarities, CCR6^+^DN distinguished from the other three subsets by superior their ability to produce Th17 effector cytokines (*e.g.,* IL-17F, IL-8, and IL-21) and their predominant frequency/counts in the blood and lymph nodes HIV-infected individuals receiving ART. Finally, we demonstrate that CCR6^+^DN harbor replication-competent HIV-DNA. Thus, we reveal the existence in humans of four Th17-polarized CCR6^+^ subsets that represent distinct stages of Th17 differentiation, with CCR6^+^DN being the most predominant and contributing to HIV reservoir persistence under ART

In summary, we demonstrated the existence in humans of four distinct Th17-polarized CCR6^+^ CD4^+^ T-cell subsets with differential expression of CCR4 and CXCR3, including two newly characterized CCR6^+^DN and CCR6^+^DP populations (Fig. 10). The existence of multiple Th17 subsets with distinct trafficking potential and functional properties might allow defenses at barrier surfaces including the gut, while maintaining a pool of long-lived CM Th17 cells, *i.e.,* CCR6^+^DN, that recirculate across lymph nodes. HIV-1 appears to exploit this heterogeneity for its replication and persistence. Considering alterations in the Th17 pool during early acute phases of HIV-1 infection [83], together with the long-lived properties of some Th17 cells [25, 28, 29], new Th17-specific therapeutic strategies are needed to prevent HIV reservoir establishment during primary infection and to induce latency reversal during the chronic phase. Outside the HIV field, this work provides a new original understanding of the functional heterogeneity of Th17 cells at molecular level that is critical for designing new therapeutic strategies to manipulate cellular features during other pathological conditions associated with impaired or exacerbated Th17 responses.

## Methods

### Study individuals and biological samples

Human individuals were recruited at the Montreal Chest Institute, McGill University Health Centre, and the Universite de Montreal Hospital Centre (CHUM), Montreal, Quebec. Peripheral blood mononuclear cells (PBMCs) were collected by leukapheresis and cryopreserved until use [15]. Cytomegalovirus (CMV) infection was determined upon detection of CMV-specific Abs using chemiluminescent microparticle immunoassay (CMIA) [84]. In parallel, matched blood and inguinal lymph node samples were collected from three HIV-infected individuals from the SCOPE study (UCSF School of Medicine, CA, USA) who were on stable ART with undetectable viremia (<40 HIV RNA copies/ml) for at least 8 years. After careful removal of the surrounding fatty tissue, lymph nodes were mechanically disrupted using frosted glass slides. The single cell suspension obtained was filtered and benzonase digested (25 U/mL) before to be used for flow cytometry analysis.

### Antibodies and polychromatic flow cytometry analysis

Detailed description available in Online supplemental material.

### Magnetic (MACS) and fluorescence activated cell sorting (FACS)

Total CD4^+^ T-cells were sorted from PBMCs by negative selection using magnetic beads (Miltenyi Biotec) [15], and stained with CD45RA, CCR4, CXCR3 and CCR6 Abs, and a cocktail of FITC–conjugated CD8, CD19, and CD56 Abs. Memory CD4^+^ T-cells (CD45RA^−^) with differential expression of CCR6, CCR4 and/or CXCR3 were sorted by FACS (BDAria II; BD Biosciences): CXCR3^−^CCR4^+^ (CCR4^+^Th17 [5, 15]), CXCR3^+^CCR4^−^ (CXCR3^+^Th1Th17 [5, 15]), CXCR3^−^CCR4^−^ (CCR6^+^DN), and CXCR3^−^CCR4^−^ (CCR6^+^DP). Total CCR6^−^ or CXCR3^+^CCR4^−^ (Th1 [5, 15]) subsets were sorted in parallel. Sorting gates were set on FITC- to exclude CD8^+^ T-cells, CD19^+^ B-cells, and CD56^+^ NK-cells. For specific experiments, central (CM, CD45RA^−^CCR7^+^) and effector memory (EM, CD45RA^−^CCR7^−^) subsets with differential expression of CCR6, CCR4 and/or CXCR3 were sorted. Sorting gates were set based on FMO controls [15]. Quality control analysis post-sort indicated an average purity >95 % (Additional file 1: S1 Figure).

### Genome-wide transcription profiling

Matched memory CD4^+^ T-cell subsets were isolated by FACS from different HIV-uninfected donors stimulated with immobilized CD3 and soluble CD28 (1 µg/ml) for 4 days. Total RNA was isolated using RNeasy columns kit (Qiagen) according to the manufacturer’s protocol. RNA quantity was determined by Pearl nanophotometer (Implen, Germany) (10^6^ cells yielded 1–5 µg RNA). Genome-wide analysis of gene expression was performed on total RNA by Génome Québec Innovation Centre (Montreal, Qc, Canada). Briefly, the quality of total RNA was tested using the Agilent 2100 Bioanalyzer chip. High quality RNA was reverse transcribed and hybridized on the Illumina HumanHT-12 v4 Expression BeadChip providing coverage for more then 47,000 transcripts and known splice variants across the human transcriptome. The expression of differentially expressed genes was identified as previously described [55]. The entire microarray dataset and technical information requested by Minimum Information About a Microarray Experiment (MIAME) are available at the Gene Expression Omnibus (GEO) database under accession number GSE66972. Differentially expressed genes (cut-off 1.3-fold; p < 0.05) were classified through Gene Ontology using the NetAffx web-based application (Affymetrix), while differentially expressed pathways were identified using *Ingenuity Pathway Analysis* (IPA) and *Gene Set Variation Analysis* (GSVA). Corresponding heat maps for biological function categories were generated using programming language R [55].

### Real-time RT-PCR

One step SYBR Green real-time RT-PCR was carried out in a LightCycler 480 II (Roche) using Qiagen reagents according to manufacturer’s recommendations, as previously described [55, 85]. Briefly, STAT3, BCL-6, Lef1, and Terc primers were purchased from Qiagen (QuantiTect Primer Assay). The expression of each gene was normalized relative to the internal control 28S rRNA levels (forward 5′-CGAGATTCCTGTCCCCACTA-3′; reverse 5′-GGGGCCACCTCCTTATTCTA-3′ (IDT [85]). Samples without template or without reverse transcriptase were used as negative controls. Each RT-PCR reaction was performed in triplicata.

### Cytokine screening and ELISA quantification

Cell culture supernatants were screened for the expression of 34 cytokines using the Human Th1/Th2/Th17 Antibody Array C series (RayBiotec, Norcross, GA), as previously described [86]. Cytokine levels were quantified by ELISA using commercial kits for IL-10, IL-13, IL-17A, IL-17F, IL-21, IL-22, IFN-γ, TNF-α (Ebioscience) as well as CXCL8/IL-8 and CCL20 (R&D Systems).

### Intracellular cytokine staining

Intracellular expression of cytokines was measured by FACS [87] using the BD Cytofix/Cytoperm kit (BD Biosciences) and specific Abs (See table above). Analysis was performed using Diva and FlowJo. Polyfunctional profiles were analyzed using the SPICE (version 5.3; provided by Mario Roederer, VRC/NIAID/NIH) [50].

### Antigen presentation assay

Cell proliferation was measured using the carboxy fluoroscein succinimidyl ester (CFSE) dilution assay [87], following co-culture with antigen-loaded autologous monocyte-derived dendritic cells (MDDC). Briefly, monocytes isolated from PBMCs by negative selection using magnetic beads (Miltenyi Biotec) [86] were differentiated into MDDC in the presence of GM-CSF and IL-4 (20 ng/ml; R&D Systems). MDDCs were loaded with SEB (25 ng/ml; Toxin Technologies), CMV-pp65 peptide pool (1 μg/ml; Miltenyi) or *Candida albicans* hyphae LAM-1 [88] (25 µl protein lysate [87]) for one hour at 37 °C and cocultured with FACS-sorted CFSE-loaded T-cell subsets. MDDC:T-cell cocultures (1:4 ratio) were maintained for 5 days at 37 °C. MDDC:T-cell co-cultures were stained with CD3 (T-cell marker) and CD1c (DC marker). Proliferating T-cells were identified as cells with a CFSE^low^CD3^+^CD1c^−^ phenotype. In parallel, MDDC:T-cell co-cultures were stimulated with PMA and Ionomycin in the presence of brefeldin A and the expression of cytokines in proliferating T-cell subsets was quantified by FACS upon intracellular staining with specific anti-cytokine Abs (See table above). Intracellular expression of cytokines was quantified by FACS [87].

### Th17 *versus* Th1 polarization

Cells were stimulated via CD3/CD28 and cultured under Th17-polarizing conditions (IL-1β (10 ng/ml), IL-6 (50 ng/ml), and IL-23 (40 ng/ml), and anti-IL-4 (1 μg/ml) and anti-IFN-γ Abs (10 μg/ml) (R&D Systems)) or Th1-polarizing conditions (IL-12 (5 ng/ml) (R&D Systems) and anti-IL-4 Abs (1 μg/ml)) for 4 days. Cells were washed and plated in media containing the Th17- or Th1-polarization cocktails together with recombinant IL-2 (5 ng/ml) for 10 additional days. Polarizing cytokines were replenished every 2–3 days and cells were split to optimal density (<2 × 10^6^ cells/ml).

### HIV infection in vitro

FACS-sorted memory CCR6^+^ T-cell subsets were activated for 4 days via CD3/CD28 (1 μg/ml) and then exposed to HIV-1 ADA8 (50 ng HIV-p24 *per* 10^6^ cells) for 3 h at 37 °C. Extensive washing was performed to remove unbound HIV. Cells (10^6^ cells *per* ml) were cultured in media containing FBS (10 %) and IL-2 (5 ng/ml; R&D Systems). Cell culture supernatants were harvested at day 3 post-infection and HIV replication was measured by HIV-p24 ELISA.

### Real-time PCR quantification of integrated HIV-DNA

The quantification of integrated HIV-DNA was performed as we previously described [15, 82]. Briefly, cells were digested in a proteinase K buffer (Invitrogen), and 10^5^ cells/15 µl lysate were used *per* amplification. Integrated HIV-DNA was amplified first (12 cycles) using two outward-facing *Alu* primers and one HIV LTR primer tagged with a lambda sequence; the CD3 gene was amplified in the same reaction. The HIV and CD3 amplicons were then amplified in separate reactions (Light Cycler 480, Roche Diagnostics). The HIV-DNA was amplified using a lambda-specific primer and an inner LTR primer in the presence of two fluorescent probes specific for HIV LTR. The CD3 DNA was amplified using inner primers and two fluorescent probes specific for CD3. Amplification reactions were carried out with Light Cycler 480 Probe Master Mix (Roche) and Taq Polymerase (Invitrogen). The ACH2 cells carrying one copy of integrated HIV-DNA per cell (NIAIDS reagent program) were used as standard curve.

### HIV reactivation assay

Highly pure matched memory CD4^+^ T-cell subsets were isolated by FACS from different CI on ART individuals and stimulated with immobilized CD3 and soluble CD28 Abs (1 µg/ml) for 4 days. Cells were washed and plated in media containing recombinant IL-2 (5 ng/ml) for 9 additional days. IL-2 was replenished and cells were split to optimal density (<2 × 10^6^ cells/ml) every 3 days. At day 13, cells were stimulated with PMA and Ionomycin in the presence of Brefeldin A for 6 h. Intracellular staining was performed with cytokine-specific Abs and HIV-p24. Cell culture supernatants were collected at day 4, 7, 11, and 13 post-TCR triggering and used for the quantification of HIV-RNA and/or HIV-p24 levels.

### Real-time RT-PCR quantification for HIV-RNA

Viral RNA was isolated from cell-culture supernatants using the QIAmp viral RNA mini kit (Qiagen) according to the manufacturer’s protocol. Collected RNA was treated with DNase, reverse transcribe and then amplified for 20 cycles using primers specific for HIV-LTR (tagged with a lambda sequence) and HIV-GAG. The HIV-DNA was further amplified using a lambda-specific primer and an inner HIV-LTR primer as well as two fluorescent probes specific for HIV-LTR, using a protocol adapted from [82].

### Statistics

Statistical analyses were performed using the GraphPad Prism 5. Details are included in Figure legends.

## Additional files

10.1186/s12977-016-0293-6 Purity of flow cytometry-sorted memory CD4^+^ T-cell subsets. Total CD4^+^ T-cells were isolated from PBMCs of healthy individuals by negative selection using magnetic beads (Miltenyi). Cells were stained with a cocktail of fluorochrome-conjugated Abs and analyzed by polychromatic flow cytometry (see Supplemental Experimental Procedure). Memory (CD45RA^−^) cells lacking the lineage-specific markers CD8 (CD8^+^ T-cells), CD19 (B cells), and CD56 (NK cells) and with differential expression of CCR6, CCR4, and CXCR3 were sorted by flow cytometry (BDAria II) as follows: CCR6^+^CCR4^+^CXCR3^−^ (Th17), CCR6^+^CCR4^−^CXCR3^−^ (CCR6^+^DN), CCR6^+^CCR4^+^CXCR3^+^ (CCR6^+^DP), CCR6^+^CCR4^−^CXCR3^+^ (Th1Th17) and CCR6^−^CCR4^−^CXCR3^+^ (Th1). A viability staining was used to exclude dead cells. The positivity gates where defined based on fluorescence minus one (FMO) controls. Shown is **(A)** the gating strategy for the identification of different subsets on CD4^+^ T-cells sorted by MACS and **(B)** purity upon FACS sorting of different memory T-cell subsets. The percentage of each subset is indicated on the figures. Results are from one donor representative of experiments performed with cells from >10 different donors. The positivity gates where defined based on fluorescence minus one (FMO) controls.
10.1186/s12977-016-0293-6 Genome-wide transcriptional profiling of the newly identified CCR6^+^DN and CCR6^+^DP subsets relative to the previously characterized Th17/CCR4^+^CCR6^+^ cells. See attached document.
10.1186/s12977-016-0293-6 GO classification of differentially expressed genes in 3 CCR6^+^ subsets. (**A-B**) Shown are Venn diagram representation of GSVA-generated canonical pathways (**A**) and biological functions (**B**), illustrating the number of differentially expressed pathways common or unique between Th17, CCR6^+^DN, and CCR6^+^DP. (**C-F**) Further, differentially expressed genes between (p < 0.05, FC cut-off 1.3) were classified based on their biological functions using *Gene Ontology* (GO) as follows: (**C**) Cell migration (**D**) Chemotaxis (**E**) Cell differentiation, and (**F**) Transcription factors. The heatmaps were generated using the R programming language and the heatmap and ggplot2 libraries (R Core Team). For each heat map, genes up and down regulated in different subsets are represented in red and blue, respectively.
10.1186/s12977-016-0293-6 Superior Th17-lineage commitment of CM *versus* EM Th17 and Th1Th17-subsets. FACS-sorted Th17, Th1Th17 and Th1 subsets with CM (CD45RA^−^CCR7^+^) and EM (CD45RA^−^CCR7^−^) phenotype were cultured for 14 days under Th17-polarizing conditions, as in Fig. 5. Shown are the statistical analyses of the intracellular expression of IL-17A, IL-17F, IL-22, IFN-γ and TNF-α by distinct Th17-polarized CM (black bars) *versus* EM (white bars) Th17, Th1Th17 and Th1 subsets. Results (mean ± SEM) were generated with matched samples from n = 3 different subjects. Paired t-test *p*-values are indicated in the figures (CM *versus* EM).
10.1186/s12977-016-0293-6 Flow cytometry analysis of cytokine co-expression at single-cell level by CM subsets upon long-term culture under Th17 versus Th1 polarizing conditions. Central memory (CM) Th17, Th1Th17, CCR6^+^DN, CCR6^+^DP, and Th1 subsets were sorted and cultured for 14 days under Th17 or Th1 polarizing conditions, as described in Fig. 5. Shown are flow cytometry dot plots illustrating the co-expression of IL-17A with IL-17F, IL-22, IFN-γ or TNF-α for each Th17- or Th1-polarized subset. Results are from one donor representative of results generated with cells from three different donors. The positivity gates where defined based on fluorescence minus one (FMO) controls.
10.1186/s12977-016-0293-6 Poly-functional profiles of CM CCR6^+^ subsets upon long-term culture under Th17 *versus* Th1 polarizing conditions in vitro. FACS-sorted CM subsets were analyzed for the expression of lineage-specific cytokines upon Th17/Th1-polarization in vitro (Fig. 5). Shown are bar graph representations generated with SPICE software for all possible combinations of one (blue), two (green), three (orange), four (yellow), and five (red) or no (purple) cytokines produced by T-cell subsets upon culture in vitro (mean ± SEM), together with pie charts summarizing the polyfunctional profiles (median relative contribution, n = 3).
10.1186/s12977-016-0293-6 Phenotypic stability *versus* plasticity of the four central memory CCR6^+^ T-cell subsets under Th17- or Th1-polarizing conditions. FACS-sorted CM subsets were cultured under Th17- and Th1-polarizing conditions for 14 days, as described in Fig. 5A, stained with CCR6, CCR4, and CXCR3Abs and analyzed by FACS. Shown are the statistical analyses for the expression of (**A**) CCR6 (**B**) CCR4, and (**C**) CXCR3, and the co-expression of (**D**) CCR6 and CCR4 (R6^+^R4^+^ phenotype) and (**E**) CCR6 and CXCR3 (R6^+^X3^+^ phenotype) on each subsets at day 0 (white bars) *versus* day 14 in culture under Th17- (black bars) and Th1-polarizing (grey bars) conditions. **(A-E)** The positivity gates where defined based on fluorescence minus one (FMO) controls. Results (mean ± SEM) were generated with matched subsets from n = 3 distinct subjects. Paired t-Test *p*-values are indicated on the graphs.
10.1186/s12977-016-0293-6 **Table S2.** Clinical parameters of chronically HIV-infected subjects under long-term viral suppressive ART (CI on ART). **Table S3.** Clinical parameters of the longitudinal cohort of HIV-infected subjects.
10.1186/s12977-016-0293-6 The four CCR6^+^ T-cell subsets isolated from HIV-infected subjects receiving ART preserve their Th17-polarizing profiles upon long term culture in vitro. **(A)** FACS-sorted Th17, Th1Th17 and Th1 subsets were stimulated with CD3/CD28 for four days then cultured in the presence of IL-2 (5 ng/ml) for an additional 9 days. At day 13, cells were stimulated with PMA and Ionomycin in the presence of Brefeldin A for 6 h. Intracellular staining was performed with cytokine-specific Abs. (**B**) Shown are flow cytometry dot plots illustrating the co-expression of IL-17A and IFNγ (n = 3). (**C**) Shown is the frequency of cytokine-expressing T-cells cultured long-term in vitro. Results (mean ± SEM) were generated with matches samples from n = 3 different donors. Paired t-Test *p*-values are indicated on the figures.
10.1186/s12977-016-0293-6 All four CCR6^+^ T-cell subsets carry integrated HIV-DNA in viremic untreated HIV-infected subjects. The four memory CCR6^+^ subsets as well as naive cells from PBMCs of (**A**) HIV^+^ recently infected untreated (RI) (n = 3) and were sorted by FACS. Levels of integrated HIV-DNA were quantified by nested real-time PCR (mean ± SD of triplicate wells).
10.1186/s12977-016-0293-6 Poly-functional profiles of HIV-p24^+^ CCR6^+^ T-cell subsets of HIV-infected individuals receiving ART upon viral reactivation in vitro. HIV reservoir reactivation was performed as described in Suppl. Figure 6 legend. Briefly, at day 13, cells were stimulated with PMA/Ionomycin in the presence of Brefeldin A and intracellular staining was performed with cytokine (IFN-γ, IL-17A, IL-22, TNF-α) and HIV-p24 Abs. Shown are bar graph representations generated with SPICE software for all possible combinations of one (blue), two (green), three (orange), and four (yellow), or no (purple) cytokines produced by HIV-p24^+^ Th17, Th1Th17, CCR6^+^DN, CCR6^+^DP and Th1 subsets (n = 3 CI on ART subjects). In contrast to CI on ART 5 and 6, for donor CI on ART 07 HIV reactivation was observed only for CCR6^+^DN (red).
