# Supplementary material for: New insights into the heterogeneity of Th17 subsets contributing to HIV-1 persistence during antiretroviral therapy
Source: Retrovirology. 2016 Aug 24;13(1):59. doi: 10.1186/s12977-016-0293-6 (PMC4995622; doi:10.1186/s12977-016-0293-6)
Supplement: Supplementary file 1 — 10.1186/s12977-016-0293-6 Purity of flow cytometry-sorted memory CD4+ T-cell subsets. Total CD4+ T-cells were isolated from PBMCs of healthy individuals by negative selection using magnetic beads (Miltenyi). Cells were stained with a cocktail of fluorochrome-conjugated Abs and analyzed by polychromatic flow cytometry (see Supplemental Experimental Procedure). Memory (CD45RA−) cells lacking the lineage-specific markers CD8 (CD8+ T-cells), CD19 (B cells), and CD56 (NK cells) and with differential expression of CCR6, CCR4, and CXCR3 were sorted by flow cytometry (BDAria II) as follows: CCR6+CCR4+CXCR3− (Th17), CCR6+CCR4−CXCR3− (CCR6+DN), CCR6+CCR4+CXCR3+ (CCR6+DP), CCR6+CCR4−CXCR3+ (Th1Th17) and CCR6−CCR4−CXCR3+ (Th1). A viability staining was used to exclude dead cells. The positivity gates where defined based on fluorescence minus one (FMO) controls. Shown is (A) the gating strategy for the identification of different subsets on CD4+ T-cells sorted by MACS and (B) purity upon FACS sorting of different memory T-cell subsets. The percentage of each subset is indicated on the figures. Results are from one donor representative of experiments performed with cells from >10 different donors. The positivity gates where defined based on fluorescence minus one (FMO) controls. [file 12977_2016_293_MOESM1_ESM.ppt]

## Slide 1
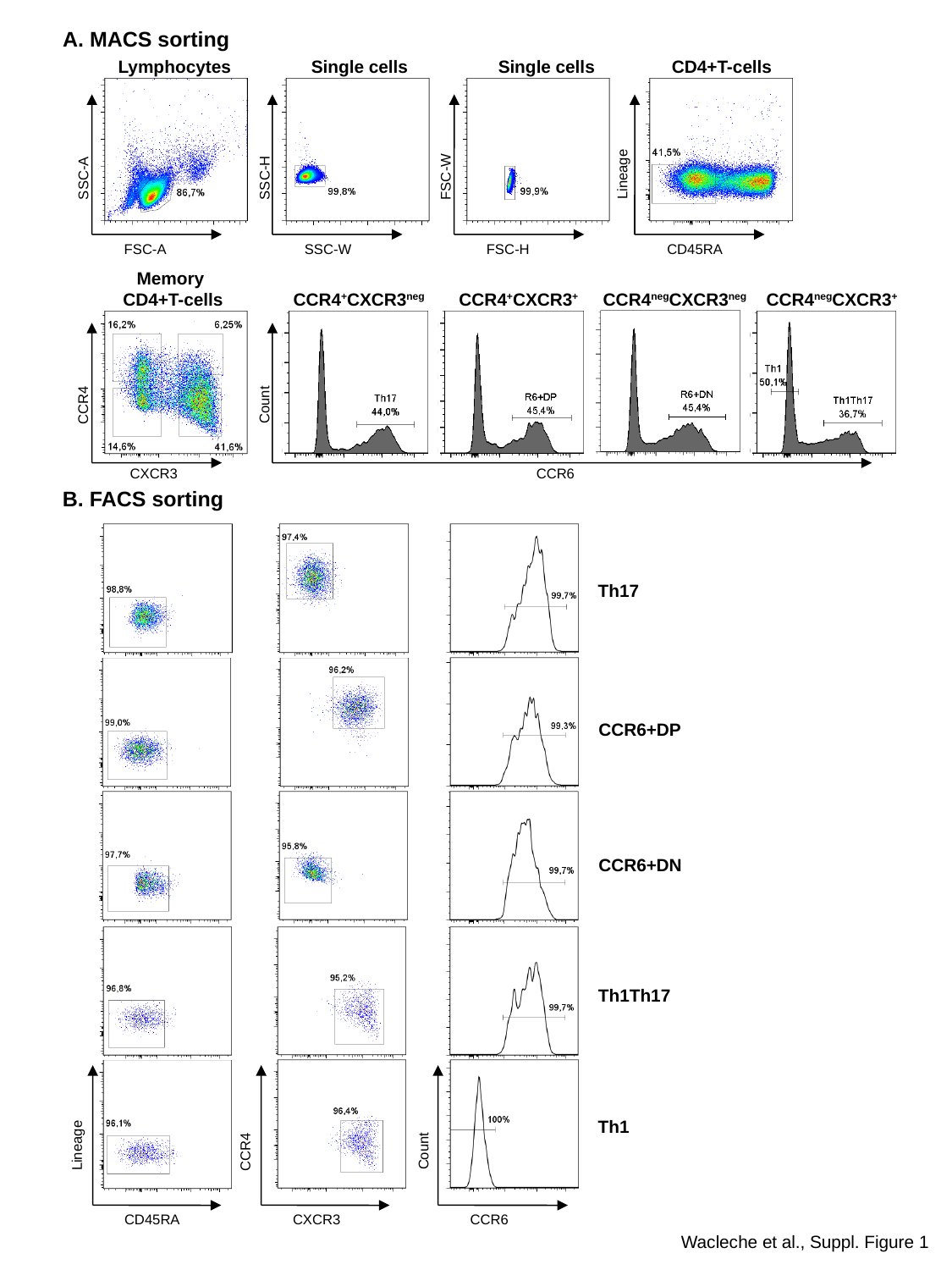

A. MACS sorting
Lymphocytes
Single cells
Single cells
CD4+T-cells
Lineage
FSC-W
SSC-H
SSC-A
FSC-A
SSC-W
FSC-H
CD45RA
Memory
CD4+T-cells
CCR4+CXCR3neg
CCR4+CXCR3+
CCR4negCXCR3neg
CCR4negCXCR3+
CCR4
Count
CXCR3
CCR6
B. FACS sorting
Th17
CCR6+DP
CCR6+DN
Th1Th17
Lineage
CD45RA
CCR4
CXCR3
Count
CCR6
Th1
Wacleche et al., Suppl. Figure 1
