# Supplementary material for: New insights into the heterogeneity of Th17 subsets contributing to HIV-1 persistence during antiretroviral therapy
Source: Retrovirology. 2016 Aug 24;13(1):59. doi: 10.1186/s12977-016-0293-6 (PMC4995622; doi:10.1186/s12977-016-0293-6)
Supplement: Supplementary file 3 — 10.1186/s12977-016-0293-6 GO classification of differentially expressed genes in 3 CCR6+ subsets. (A-B) Shown are Venn diagram representation of GSVA-generated canonical pathways (A) and biological functions (B), illustrating the number of differentially expressed pathways common or unique between Th17, CCR6+DN, and CCR6+DP. (C-F) Further, differentially expressed genes between (p < 0.05, FC cut-off 1.3) were classified based on their biological functions using Gene Ontology (GO) as follows: (C) Cell migration (D) Chemotaxis (E) Cell differentiation, and (F) Transcription factors. The heatmaps were generated using the R programming language and the heatmap and ggplot2 libraries (R Core Team). For each heat map, genes up and down regulated in different subsets are represented in red and blue, respectively. [file 12977_2016_293_MOESM3_ESM.ppt]

## Slide 1
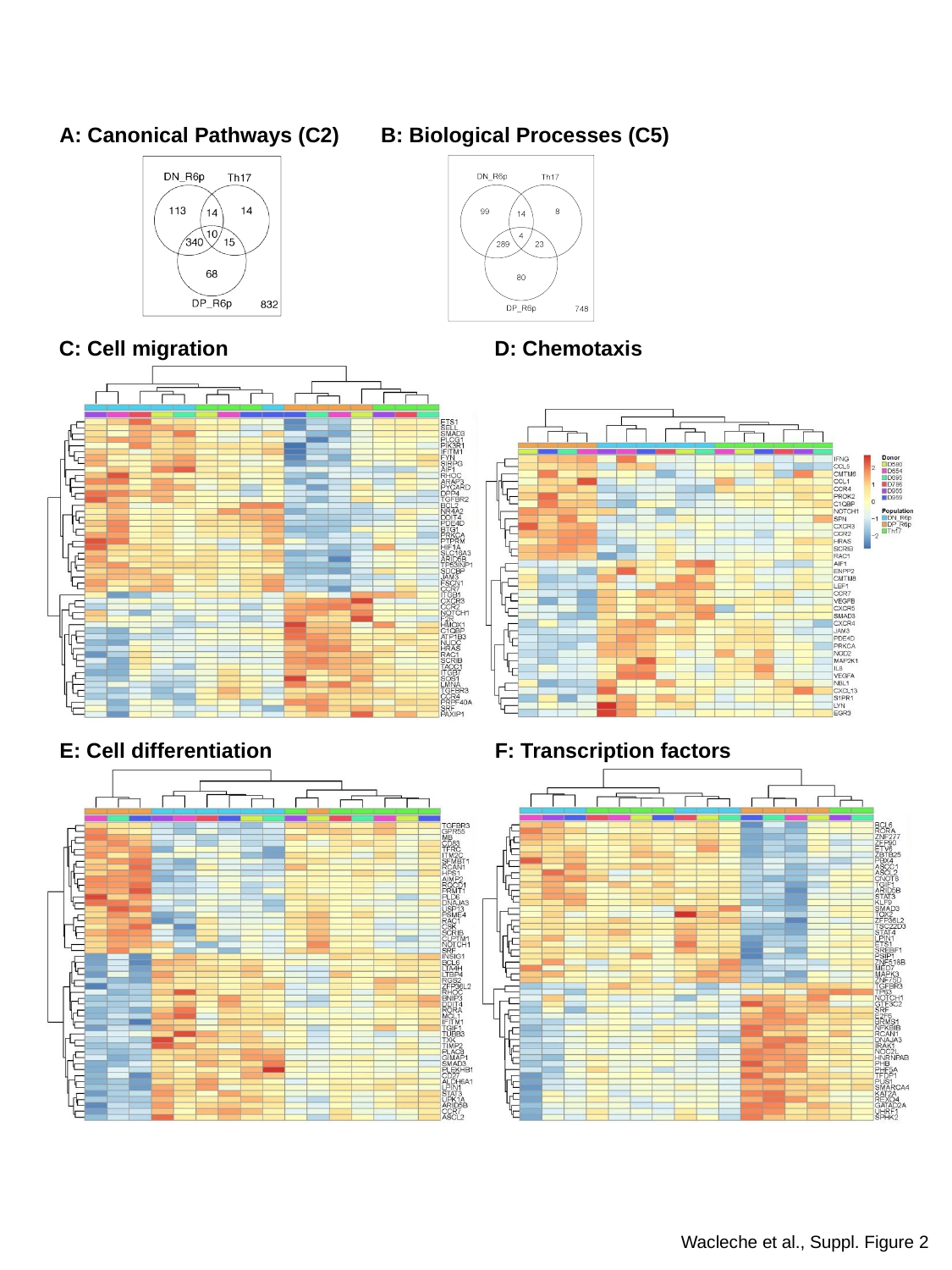

A: Canonical Pathways (C2)
B: Biological Processes (C5)
C: Cell migration
D: Chemotaxis
E: Cell differentiation
F: Transcription factors
Wacleche et al., Suppl. Figure 2
