# Supplementary figures and images for: New insights into the heterogeneity of Th17 subsets contributing to HIV-1 persistence during antiretroviral therapy
Source: Retrovirology. 2016 Aug 24;13(1):59. doi: 10.1186/s12977-016-0293-6 (PMC4995622; doi:10.1186/s12977-016-0293-6)

## Slide 1
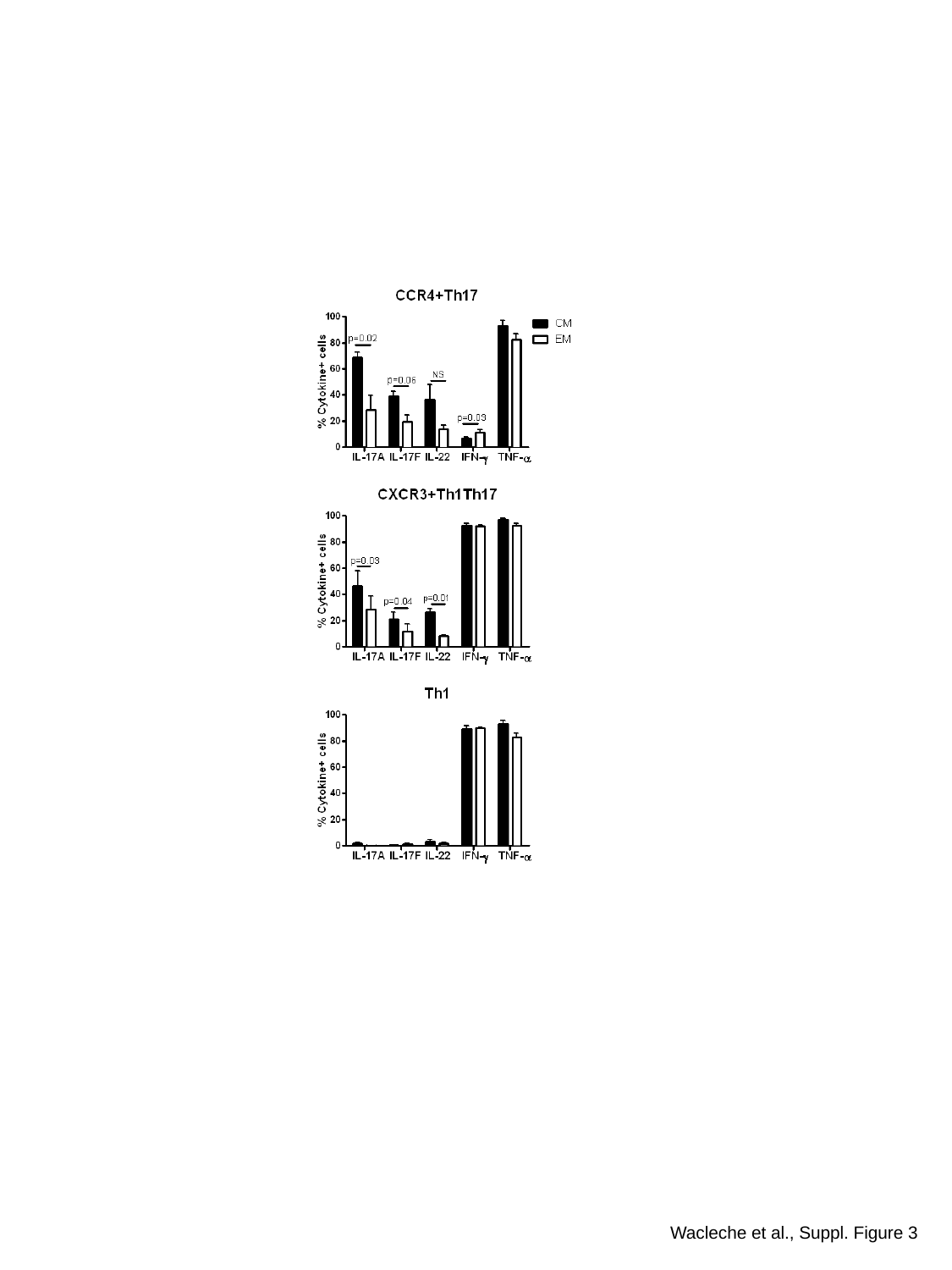

Wacleche et al., Suppl. Figure 3

Supplement: Supplementary file 4 — 10.1186/s12977-016-0293-6 Superior Th17-lineage commitment of CM versus EM Th17 and Th1Th17-subsets. FACS-sorted Th17, Th1Th17 and Th1 subsets with CM (CD45RA−CCR7+) and EM (CD45RA−CCR7−) phenotype were cultured for 14 days under Th17-polarizing conditions, as in Fig. 5. Shown are the statistical analyses of the intracellular expression of IL-17A, IL-17F, IL-22, IFN-γ and TNF-α by distinct Th17-polarized CM (black bars) versus EM (white bars) Th17, Th1Th17 and Th1 subsets. Results (mean ± SEM) were generated with matched samples from n = 3 different subjects. Paired t-test p-values are indicated in the figures (CM versus EM). [file 12977_2016_293_MOESM4_ESM.ppt]

## Slide 1
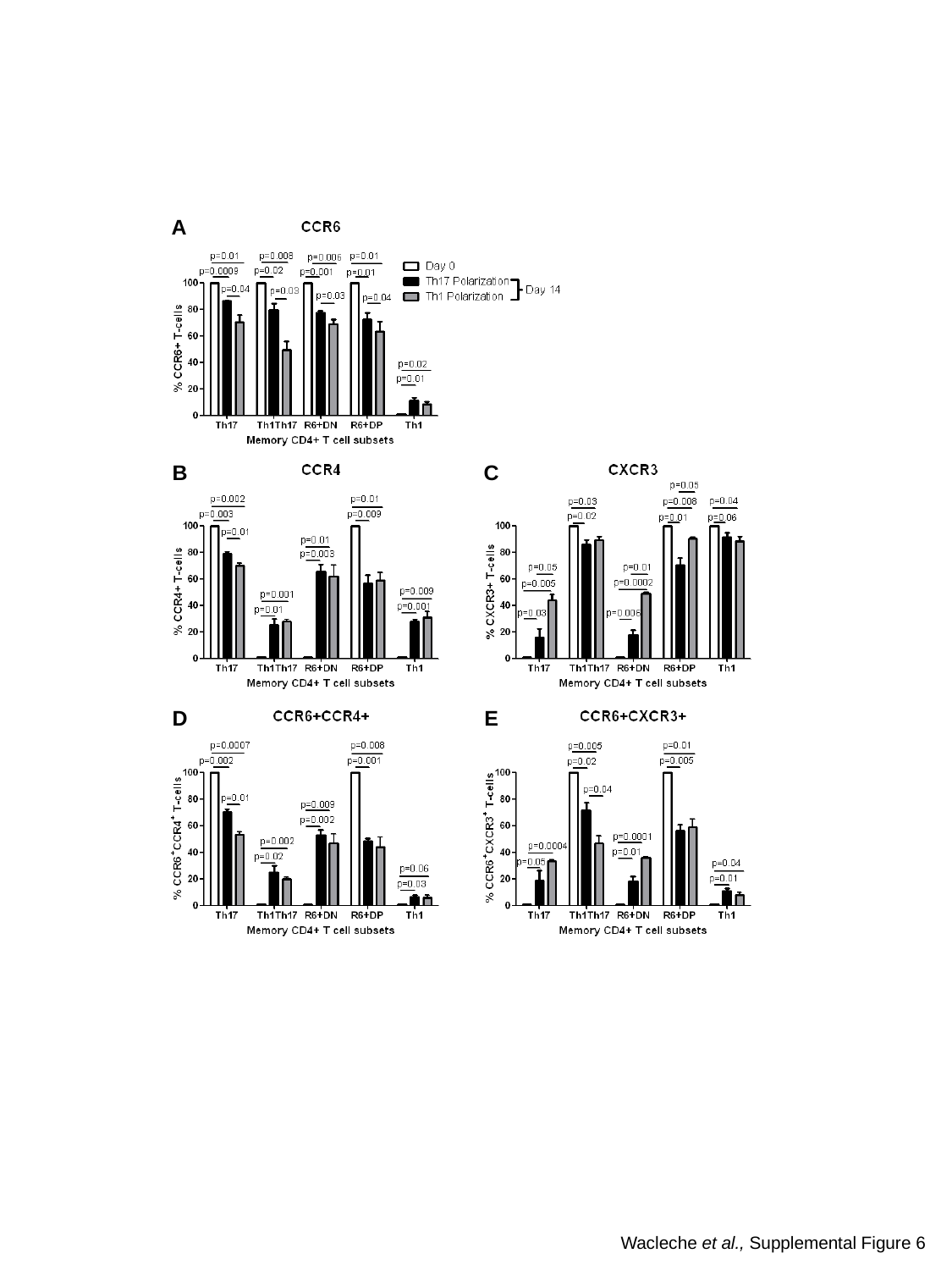

A
B
C
D
E
Wacleche et al., Supplemental Figure 6

Supplement: Supplementary file 7 — 10.1186/s12977-016-0293-6 Phenotypic stability versus plasticity of the four central memory CCR6+ T-cell subsets under Th17- or Th1-polarizing conditions. FACS-sorted CM subsets were cultured under Th17- and Th1-polarizing conditions for 14 days, as described in Fig. 5A, stained with CCR6, CCR4, and CXCR3Abs and analyzed by FACS. Shown are the statistical analyses for the expression of (A) CCR6 (B) CCR4, and (C) CXCR3, and the co-expression of (D) CCR6 and CCR4 (R6+R4+ phenotype) and (E) CCR6 and CXCR3 (R6+X3+ phenotype) on each subsets at day 0 (white bars) versus day 14 in culture under Th17- (black bars) and Th1-polarizing (grey bars) conditions. (A-E) The positivity gates where defined based on fluorescence minus one (FMO) controls. Results (mean ± SEM) were generated with matched subsets from n = 3 distinct subjects. Paired t-Test p-values are indicated on the graphs. [file 12977_2016_293_MOESM7_ESM.ppt]

## Slide 1
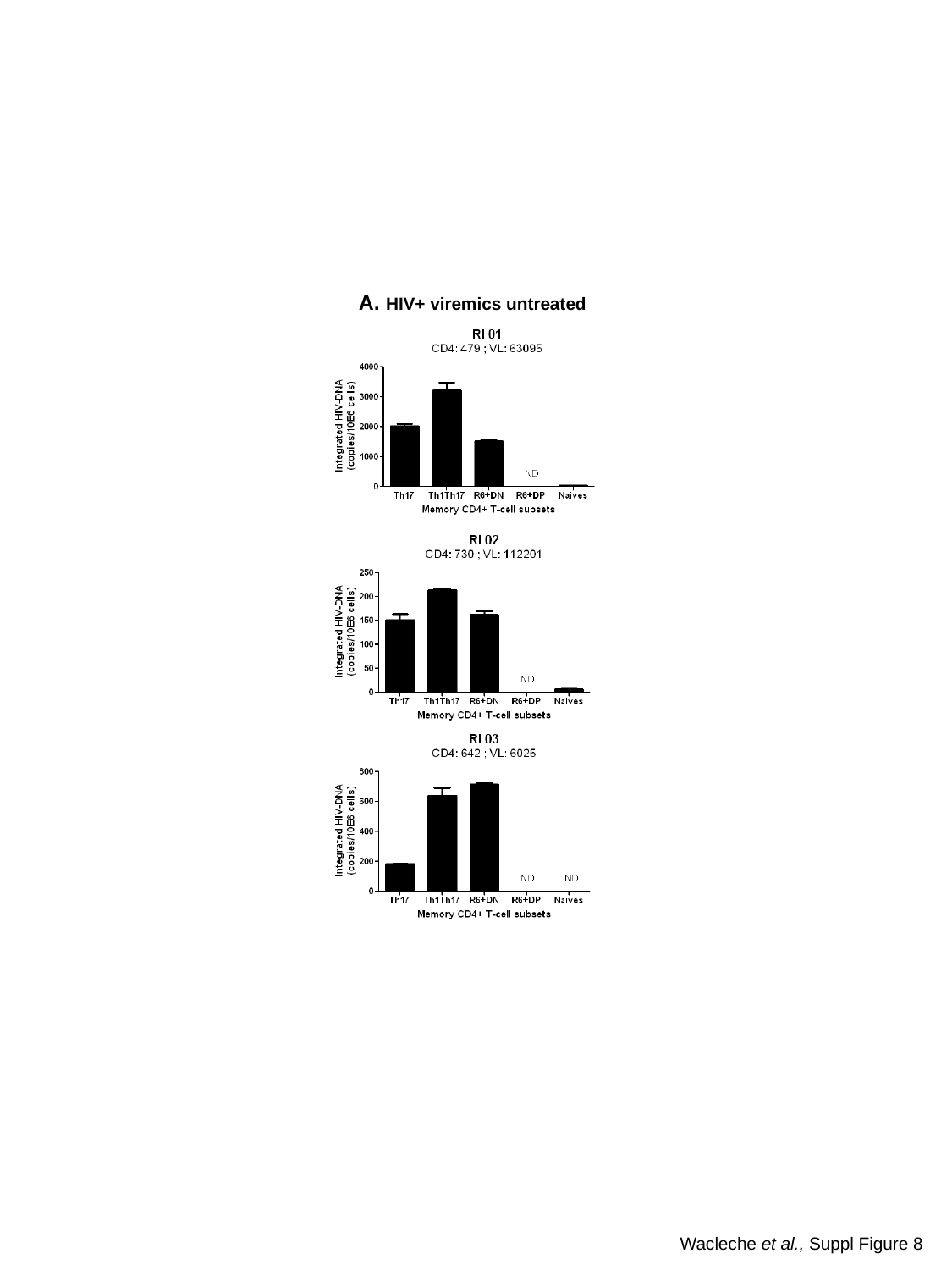

A. HIV+ viremics untreated
Wacleche et al., Suppl Figure 8

Supplement: Supplementary file 10 — 10.1186/s12977-016-0293-6 All four CCR6+ T-cell subsets carry integrated HIV-DNA in viremic untreated HIV-infected subjects. The four memory CCR6+ subsets as well as naive cells from PBMCs of (A) HIV+ recently infected untreated (RI) (n = 3) and were sorted by FACS. Levels of integrated HIV-DNA were quantified by nested real-time PCR (mean ± SD of triplicate wells). [file 12977_2016_293_MOESM10_ESM.ppt]

## Slide 1
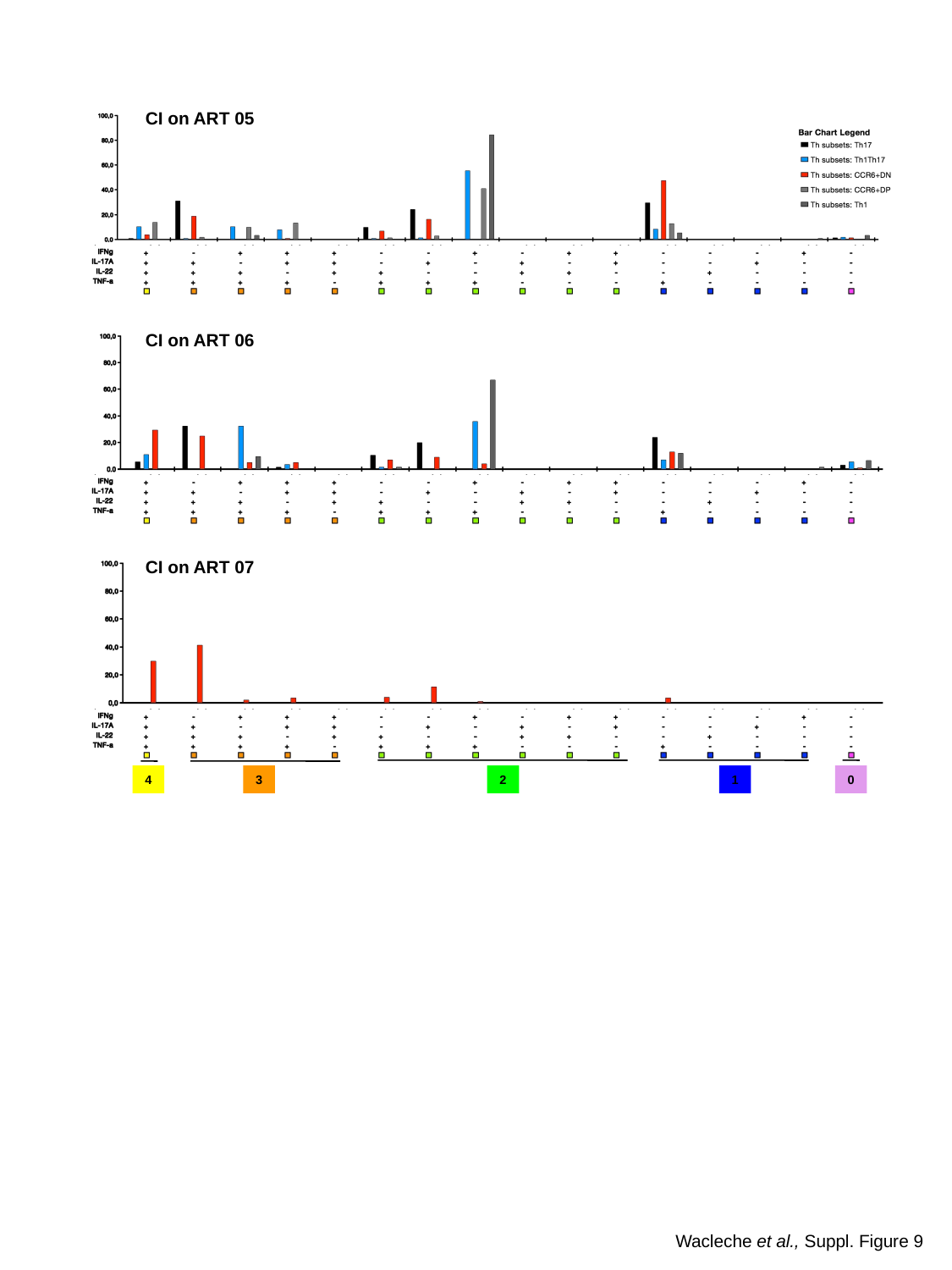

CI on ART 05
CI on ART 06
CI on ART 07
4
3
2
1
0
Wacleche et al., Suppl. Figure 9

Supplement: Supplementary file 11 — 10.1186/s12977-016-0293-6 Poly-functional profiles of HIV-p24+ CCR6+ T-cell subsets of HIV-infected individuals receiving ART upon viral reactivation in vitro. HIV reservoir reactivation was performed as described in Suppl. Figure 6 legend. Briefly, at day 13, cells were stimulated with PMA/Ionomycin in the presence of Brefeldin A and intracellular staining was performed with cytokine (IFN-γ, IL-17A, IL-22, TNF-α) and HIV-p24 Abs. Shown are bar graph representations generated with SPICE software for all possible combinations of one (blue), two (green), three (orange), and four (yellow), or no (purple) cytokines produced by HIV-p24+ Th17, Th1Th17, CCR6+DN, CCR6+DP and Th1 subsets (n = 3 CI on ART subjects). In contrast to CI on ART 5 and 6, for donor CI on ART 07 HIV reactivation was observed only for CCR6+DN (red). [file 12977_2016_293_MOESM11_ESM.ppt]
