# Supplementary material for: New insights into the heterogeneity of Th17 subsets contributing to HIV-1 persistence during antiretroviral therapy
Source: Retrovirology. 2016 Aug 24;13(1):59. doi: 10.1186/s12977-016-0293-6 (PMC4995622; doi:10.1186/s12977-016-0293-6)
Supplement: Supplementary file 5 — 10.1186/s12977-016-0293-6 Flow cytometry analysis of cytokine co-expression at single-cell level by CM subsets upon long-term culture under Th17 versus Th1 polarizing conditions. Central memory (CM) Th17, Th1Th17, CCR6+DN, CCR6+DP, and Th1 subsets were sorted and cultured for 14 days under Th17 or Th1 polarizing conditions, as described in Fig. 5. Shown are flow cytometry dot plots illustrating the co-expression of IL-17A with IL-17F, IL-22, IFN-γ or TNF-α for each Th17- or Th1-polarized subset. Results are from one donor representative of results generated with cells from three different donors. The positivity gates where defined based on fluorescence minus one (FMO) controls. [file 12977_2016_293_MOESM5_ESM.ppt]

## Slide 1
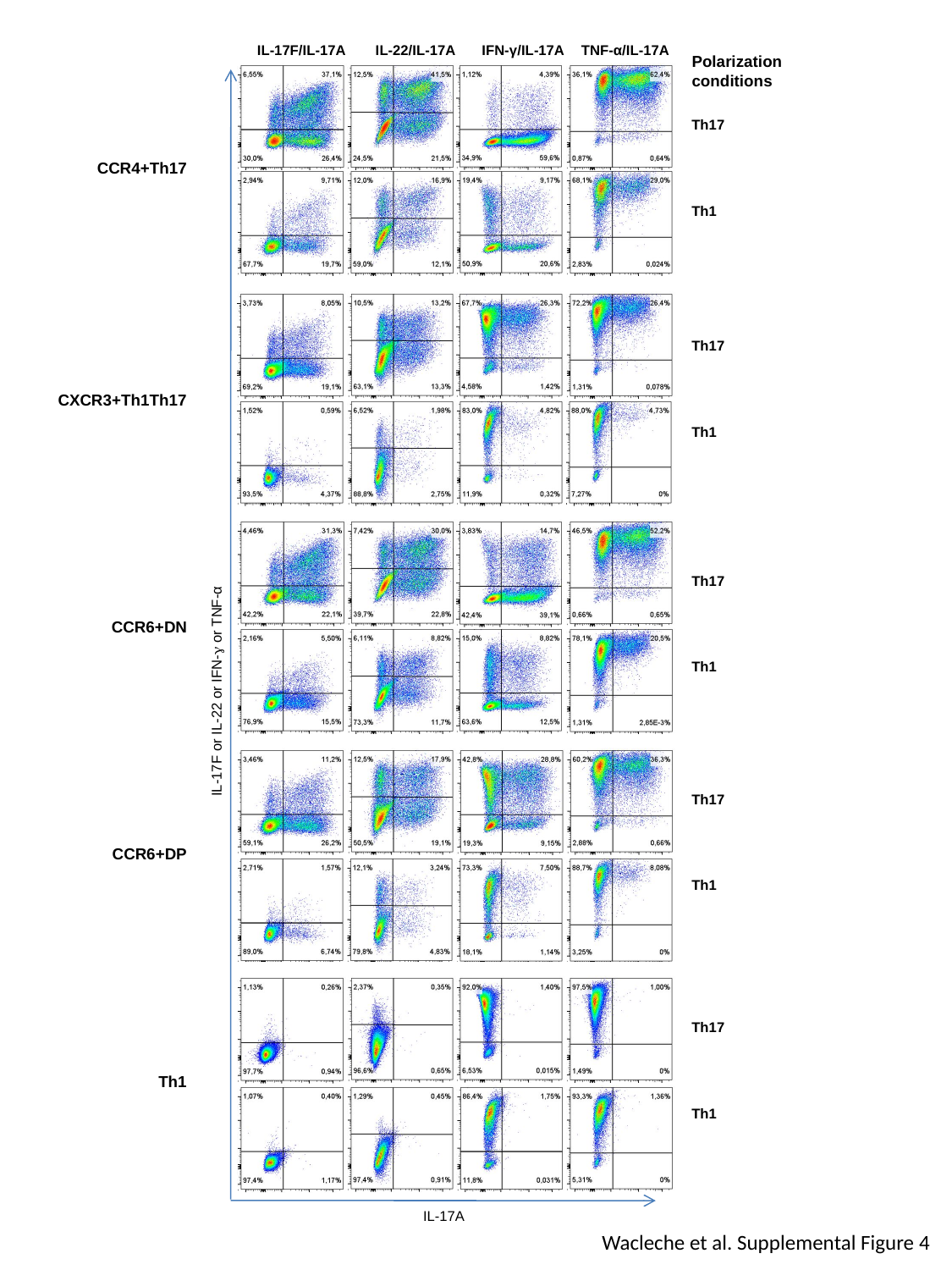

IL-17F/IL-17A
IL-22/IL-17A
IFN-γ/IL-17A
TNF-α/IL-17A
Polarization
conditions
Th17
CCR4+Th17
Th1
Th17
CXCR3+Th1Th17
Th1
Th17
CCR6+DN
Th1
IL-17F or IL-22 or IFN-γ or TNF-α
Th17
CCR6+DP
Th1
Th17
Th1
Th1
IL-17A
Wacleche et al. Supplemental Figure 4
