# Supplementary material for: New insights into the heterogeneity of Th17 subsets contributing to HIV-1 persistence during antiretroviral therapy
Source: Retrovirology. 2016 Aug 24;13(1):59. doi: 10.1186/s12977-016-0293-6 (PMC4995622; doi:10.1186/s12977-016-0293-6)
Supplement: Supplementary file 6 — 10.1186/s12977-016-0293-6 Poly-functional profiles of CM CCR6+ subsets upon long-term culture under Th17 versus Th1 polarizing conditions in vitro. FACS-sorted CM subsets were analyzed for the expression of lineage-specific cytokines upon Th17/Th1-polarization in vitro (Fig. 5). Shown are bar graph representations generated with SPICE software for all possible combinations of one (blue), two (green), three (orange), four (yellow), and five (red) or no (purple) cytokines produced by T-cell subsets upon culture in vitro (mean ± SEM), together with pie charts summarizing the polyfunctional profiles (median relative contribution, n = 3). [file 12977_2016_293_MOESM6_ESM.ppt]

## Slide 1
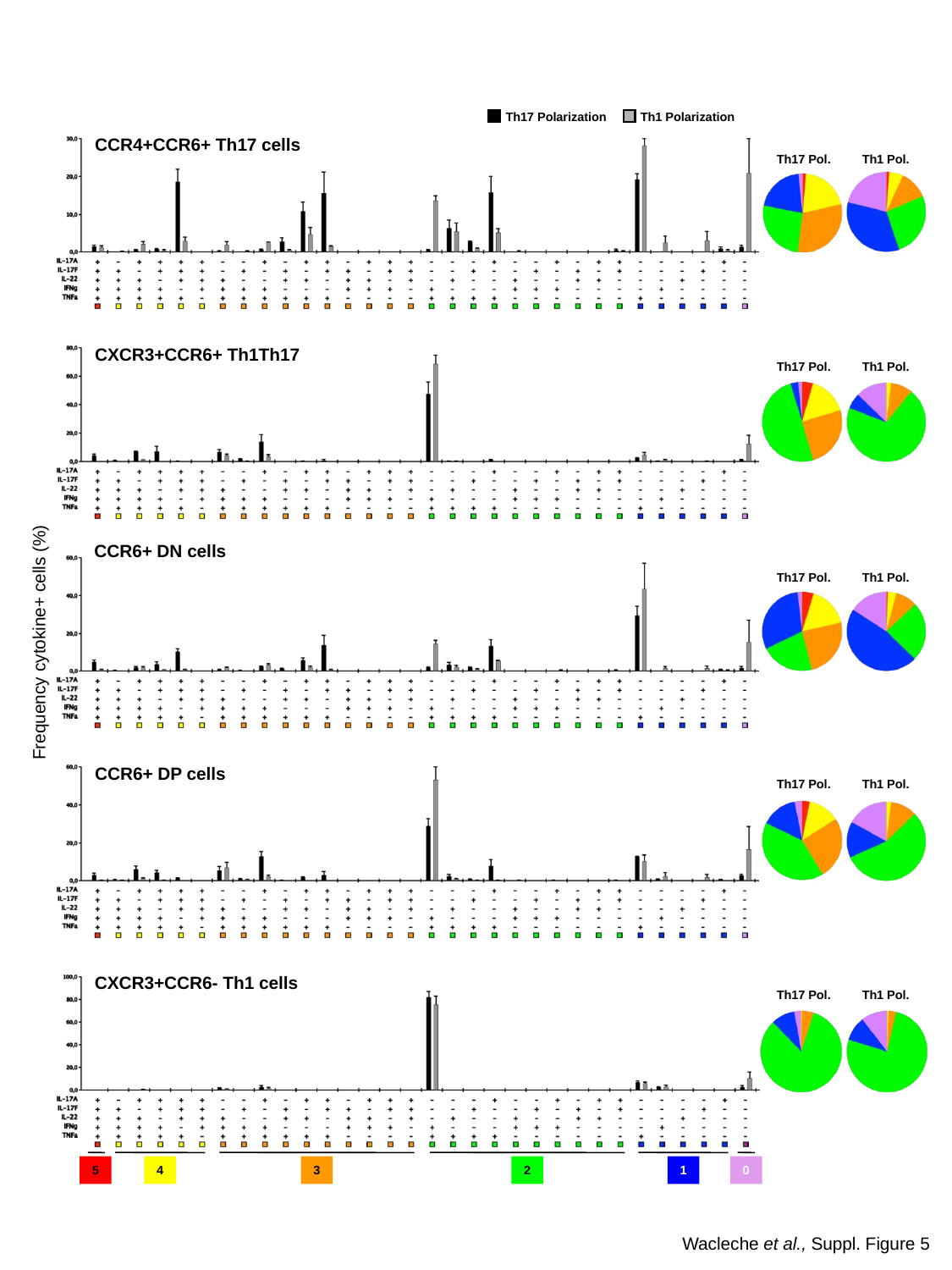

Th17 Polarization
Th1 Polarization
CCR4+CCR6+ Th17 cells
Th17 Pol.
Th1 Pol.
CXCR3+CCR6+ Th1Th17
Th17 Pol.
Th1 Pol.
CCR6+ DN cells
Th17 Pol.
Th1 Pol.
Frequency cytokine+ cells (%)
CCR6+ DP cells
Th17 Pol.
Th1 Pol.
CXCR3+CCR6- Th1 cells
Th17 Pol.
Th1 Pol.
5
4
3
2
1
0
Wacleche et al., Suppl. Figure 5
