# Supplementary material for: New insights into the heterogeneity of Th17 subsets contributing to HIV-1 persistence during antiretroviral therapy
Source: Retrovirology. 2016 Aug 24;13(1):59. doi: 10.1186/s12977-016-0293-6 (PMC4995622; doi:10.1186/s12977-016-0293-6)
Supplement: Supplementary file 8 — 10.1186/s12977-016-0293-6 Table S2. Clinical parameters of chronically HIV-infected subjects under long-term viral suppressive ART (CI on ART). Table S3. Clinical parameters of the longitudinal cohort of HIV-infected subjects. [file 12977_2016_293_MOESM8_ESM.doc]

**ONLINE SUPPLEMENTAL MATERIAL**

**SUPPLEMENTAL TABLES**

**Supplemental Table 1:** Genome-wide transcriptional profiling of the newly identified CCR6^+^DN and CCR6^+^DP subsets relative to the previously characterized Th17/CCR4^+^CCR6^+^ cells. See attached document.

**Supplemental Table 2**: Clinical parameters of chronically HIV-infected subjects under long-term viral suppressive ART (CI on ART)

| **Subjects** | **CD4 counts^#^** | **CD8 counts^#^** | **Plasma viral load^&^** | **Time since infection*** | **ART** | **Time of aviremia^$^** |
| --- | --- | --- | --- | --- | --- | --- |
| **RI 01** | 479 | 1,105 | 63,095 | 41 | none | 0 |
| **RI 02** | 730 | 520 | 112,201 | 8 | none | 0 |
| **RI 03** | 642 | 847 | 6,025 | 3 | none | 0 |
| **CI 01** | 498 | 531 | <50 | 213 | TMC114; RAL; ETR; RTV | 52 |
| **CI 02** | 543 | 787 | <50 | 214 | TDF+FTC;RTV; TPV;RAL | 66 |
| **CI 03** | 318 | 431 | <50 | 148 | DLV ; ABC+3TC | 36 |
| **CI 04** | 847 | 944 | <50 | 168 | ATV; ABC+3TC | 2 |
| **CI 05** | 581 | 1060 | <50 | 96 | EFV; TDF+FTC | N/D |
| **CI 06** | 456 | 619 | <50 | 52 | FTC+RPV+TDF | 80 |
| **CI 07** | 459 | 545 | <50 | 215 |  | N/D |
| **CI 08** | 890 | 673 | <50 | 57 | AZT ; 3TC ; NEV | 42 |
| **CI 09** | 463 | 757 | <50 | 152 | 3TC; EFV; ABA | 20 |
| **CI 10** | 602 | 767 | <50 | 158 | 3TC; ABA ; SAQ | 53 |
| **CI 11** | 563 | 613 | <50 | 86 | IND; 3TC ; AZT | 71 |
| **CI 12** | 424 | 461 | <50 | 84 | 3TC; D4T ; DLV | 46 |
| **CI 13** | 731 | 413 | <50 | 51 | EFV; AZT ; 3TC | 22 |
| **CI 14** | 834 | 527 | <50 | 38 | NEV; ATV ; TDF ; RTV | 25 |
| **CI 15** | 552 | 715 | <50 | 139 | D4T; ATV | 56 |
| **CI 16** | 671 | 1,120 | <50 | 242 | 3TC; ABA ; LOP ; RTV | 64 |
| **CI 17** | 510 | 765 | <50 | 61 | AZT; 3TC ; RTV | 52 |
| **CI 18** | 799 | 1,727 | <50 | 62 | 3TC; D4T; NEV | 33 |
| **CI 19** | 501 | 278 | <50 | 90 | D4T; 3TC; IND | 87 |
| **CI 20** | 344 | 642 | <50 | 59 | 3TC; D4T; NEV | 44 |
| **CI 21** | 604 | 1,281 | <50 | 53 | IND; AZT; 3TC | 35 |
| **CI 22** | 443 | 322 | <50 | 18 | RTV; AZT; 3TC; KAL | 12 |
| **CI 23** | 599 | 923 | <50 | 86 | AZT; 3TC; EFV | 46 |
| **CI 24** | 688 | 1,273 | <50 | 100 | AZT; 3TC; EFV | 59 |
| **CI 25** | 434 | 583 | <50 | 165 | 3TC; EFV; ABA | 34 |
| **CI 26** | 492 | 582 | <50 | 170 | RTV; ABC; 3TC; ATV | 66 |
| **CI 27** | 529 | 690 | <50 | 49 | 3TC; D4T; DLV | 11 |
| **CI 28** | 776 | 478 | <50 | 288 | FTC+TDF+EFV | 148 |
| **CI 29** | 277 | 909 | <50 | 11 | FTC+RPV+TDF | 2 |
| **CI 30** | 616 | 330 | <50 | 186 | TDF+FTC + Viracept | 14 |
| **CI 31** | 517 | 259 | <50 | 82 | ABC+3TC ; EFV | 108 |
| **CI 32** | 886 | 579 | <50 | 60 | TDF+FTC; RAL | 12 |
| **CI 33** | 569 | 462 | <50 | 111 | TMC114+RAL | 13 |
| **CI 34** | 269 | 282 | <50 | 96 | EFV; TDF+FTC | ND |
| **CI 35** | 391 | 620 | <50 | 165 | TMC114;ABC+3TC | 4 |
| **CI 36** | 730 | 741 | <50 | 312 | TDF;NFV;TCV | 173 |
| **CI 37** | 639 | 1317 | <50 | 360 | TDF+FTC, DTG | 137 |
| **CI 38** | 873 | 886 | <50 | 252 | FTC+TDF+EFV | 107 |
| **Median RI** | **642** | **520** | **63,095** | **8** | **NA** | **NA** |
| **Median CI** | **557,5** | **582,5** | **<50** | **98** | **NA** | **NA** |

^#^, cells/µl; ^&^, HIV RNA copies per ml plasma; *, months; ART, antiretroviral therapy; ^$^, months

**ART**: 3TC, Lamivudine; ABA/ABC, Abacavir; ATV, Atazanavir; AZT, Zidovudine; D4T, Stavudine; DLV, Delavirdine; TMC114, Duranavir; EFV, Efavirenz; IND, Indinavir; ETR, Etravirine; KAL, Kaletra; ABC+3TC, Kivexa; LOP, Lopinavir; NA, not available; NEV, Nevirapine; RTV, Ritonavir; SAQ, Saquinavir; RAL, Raltegravir; TDF, Tenofovir; FTC, Emtricitabine;RPV,rilpivirine ; TDF+FTC, Truvada; FTC+TDF+EFV, Atripla ; FTC+RPV+TDF, complera ; ABC+3TC, Kivexa ; NA, not applicable ; ND, not determined.

**Supplemental Table 3**: Clinical parameters of the longitudinal cohort of HIV-infected subjects

| **Subjects** | **Visit #** | **CD4 counts^#^** | **Plasma viral load^&^** | **Time since infection*** | **Time since inclusion*** | **ART** |
| --- | --- | --- | --- | --- | --- | --- |
| **HPI #1**  ETI <74 days  ART at 8 months since infection | V1 | 430 | 35,303 | 2.5 | 0 | No |
|  | V2 | 520 | 2,301 | 3 | 0.5 | No |
|  | V3 | 450 | 24,845 | 3.5 | 1 | No |
|  | V4 | 350 | 38,395 | 4 | 1.5 | No |
|  | V8 | 310 | 681 | 8.5 | 6 | Yes |
|  | V9 | 690 | 40 | 12 | 9 | Yes |
|  | V11 | 690 | 40 | 18 | 15 | Yes |
|  | V12 | - | 40 | 21 | 18 | Yes |
|  | V13 | 650 | 40 | 24 | 21 | Yes |
| **HPI #2** | V1 | 510 | 127,175 | 1.5 | 0 | No |
| ETI <47 days | V6  V7  V8  V9  V10 | 380  510  420  510  500 | 51,426  19,884  196,433  75,053  286 | 5  6  7.5  10  12.5 | 3  4  6  8  11 | No  No  No  No  Yes |
| ART at 12 months since infection |  |  |  |  |  |  |
| **HPI #3**  ETI <46 days  ART at 11 months since infection | V1 | 580 | 86,627 | 1.5 | 0 | No |
|  | V2 | 540 | 37,335 | 2 | 1 | No |
|  | V4 | 740 | 27,348 | 3 | 1,5 | No |
|  | V5 | 730 | 20,076 | 3.5 | 2 | No |
|  | V7 | 670 | 11,127 | 5 | 4 | No |
|  | V8 | 530 | 26,574 | 7.5 | 6 | No |
|  | V9 | 460 | 85,718 | 10 | 9 | No |
|  | V10 | 380 | 664 | 13.5 | 12 | Yes |
|  | V11 | 660 | 113 | 17 | 16 | Yes |
|  | V12 | 640 | 40 | 20 | 18 | Yes |
|  | V13 | 780 | 40 | 22 | 21 | Yes |
| **HPI #4**  ETI<31  ART at 4 months since infection | V1 | 240 | 29,981,000 | 1 | 0 | No |
|  | V3 | 700 | 129,717 | 2 | 1 | No |
|  | V4 | 340 | 76,626 | 2.5 | 2 | No |
|  | V7 | 650 | 712 | 5 | 4 | Yes |
|  | V11 | 670 | 40 | 16 | 15 | Yes |
|  | V12 | 870 | 40 | 20 | 18 | Yes |
|  | V13 | 830 | 40 | 23 | 21 | Yes |
| **HPI #5**  ETI<76  ART at 9.5 months since infection | V1 | 407 | 189,343 | 2.5 | 0 | No |
|  | V2 | 430 | 173,044 | 3.5 | 1 | No |
|  | V3 | 403 | 59,181 | 4 | 2 | No |
|  | V7 | 396 | 81,037 | 6.5 | 4 | No |
|  | V9 | 467 | 74 | 11 | 9 | Yes |
|  | V10 | 496 | 50 | 13 | 11 | Yes |
|  | V11 | 516 | 50 | 17 | 14 | Yes |
|  | V12 | 567 | 50 | 20.5 | 18 | Yes |

^#^, cells/µL; ^&^, HIV-RNA copies per mL plasma (log_10_); *, months; ART, antiretroviral therapy; ETI: estimated time of infection

**ONLINE SUPPLEMENTAL MATERIAL**

**Antibodies and polychromatic flow cytometry analysis**

Surface staining was performed with fluorochrome-conjugated Abs (Table below), as previously described (1). The viability dye LIVE/DEAD® Fixable Aqua Dead Cell Stain Kit (Invitrogen) was used to exclude dead cells. Cells were analyzed using a LSRII cytometer, Diva version 6 (BD Biosciences, San Jose, CA), and FlowJo version 10.0.6 (Tree Star, Inc). Positivity gates were placed using fluorescence minus one (FMO) (1, 2).

**Antibodies used for flow cytometry**

| **Antibodies** | **Fluorochrome** | **Clone** | **Vendor** |
| --- | --- | --- | --- |
| CD3 | Pacific blue | UCHT1 | BD Pharmingen, San Diego, CA |
| CD4 | Alexa Fluor 700 | RPA-T4 |
| CD45RA | Allophycocyanin/Cy7 | HI100 |
| CCR4 | PE/Cy7 | 1G1 |
| CXCR3 | PE/Cy5 | 1C6 |
| CCR6 | PE | 11A9 |
| CD161 | PE/Cy5 | DX12 |
| IFN-γ  CD27 | Alexa700  BV650 | B27  L128 |
| CCR7 | FITC | 150503 | R&D Systems, Minneapolis, MN |
| CXCR3 | FITC | 49801 |
| CD8 | FITC | BW135/80 | MiltenyiBiotec, Auburn, CA |
| CD19 | FITC | LT19 |
| CD1c/BDCA-1 | PE | AD5-8E7 |
| CD56 | FITC | MEM188 | eBioscience, San Diego, CA |
| IL-17A | PE | 64DEC17 |
| IL-17F | Alexa647 | SHLR17 |
| IL-22 | PE/Cy7 | 22URTI |
| TNF-α | Pacific Blue | MAB11 |
| HIV-p24 | PE | KC57 | Beckman Coulter,  Brea, CA |

**SUPPLEMENTAL REFERENCES**

1. Gosselin A, Monteiro P, Chomont N, Diaz-Griffero F, Said EA, Fonseca S, Wacleche V, El-Far M, Boulassel MR, Routy JP, et al: Peripheral blood CCR4+CCR6+ and CXCR3+CCR6+CD4+ T cells are highly permissive to HIV-1 infection. J Immunol 2010, 184:1604-1616.

2. Roederer M. Compensation in flow cytometry. Curr Protoc Cytom. 2002, Dec;Chapter 1:Unit 1 14.
