# Supplementary material for: New insights into the heterogeneity of Th17 subsets contributing to HIV-1 persistence during antiretroviral therapy
Source: Retrovirology. 2016 Aug 24;13(1):59. doi: 10.1186/s12977-016-0293-6 (PMC4995622; doi:10.1186/s12977-016-0293-6)
Supplement: Supplementary file 9 — 10.1186/s12977-016-0293-6 The four CCR6+ T-cell subsets isolated from HIV-infected subjects receiving ART preserve their Th17-polarizing profiles upon long term culture in vitro. (A) FACS-sorted Th17, Th1Th17 and Th1 subsets were stimulated with CD3/CD28 for four days then cultured in the presence of IL-2 (5 ng/ml) for an additional 9 days. At day 13, cells were stimulated with PMA and Ionomycin in the presence of Brefeldin A for 6 h. Intracellular staining was performed with cytokine-specific Abs. (B) Shown are flow cytometry dot plots illustrating the co-expression of IL-17A and IFNγ (n = 3). (C) Shown is the frequency of cytokine-expressing T-cells cultured long-term in vitro. Results (mean ± SEM) were generated with matches samples from n = 3 different donors. Paired t-Test p-values are indicated on the figures. [file 12977_2016_293_MOESM9_ESM.ppt]

## Slide 1
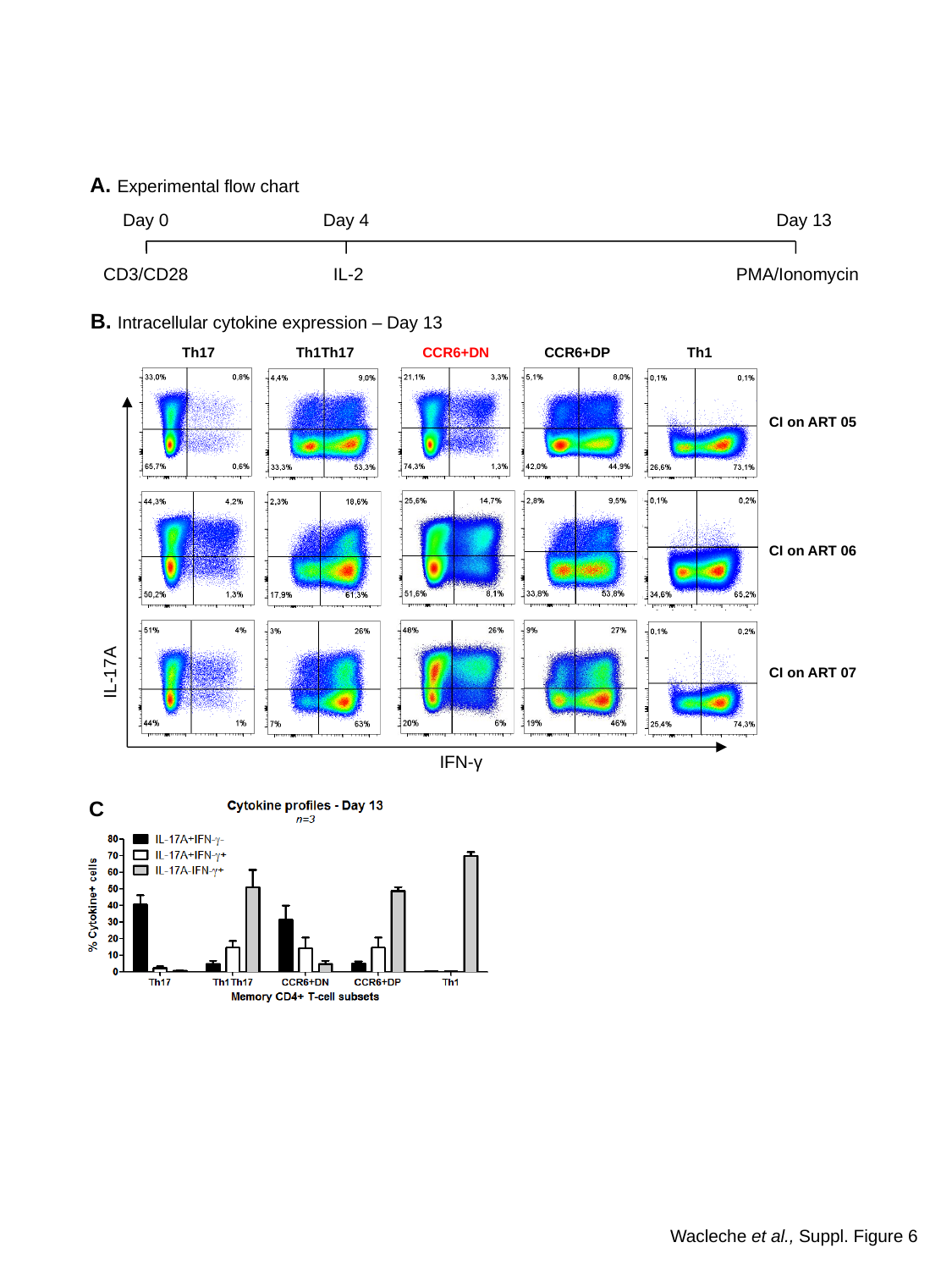

A. Experimental flow chart
Day 0
Day 4
Day 13
CD3/CD28
 IL-2
PMA/Ionomycin
B. Intracellular cytokine expression – Day 13
Th17
Th1Th17
CCR6+DN
CCR6+DP
Th1
CI on ART 05
CI on ART 06
IL-17A
CI on ART 07
IFN-γ
C
Wacleche et al., Suppl. Figure 6
